# Review of Optical Fiber and Integrated Photonic Sensors for Industry and Smart Manufacturing: Technologies, Applications, Structural Health Monitoring and AI-Enabled Sensing

**DOI:** 10.3390/s26113581

**Published:** 2026-06-04

**Authors:** Giannis Poulopoulos, Hercules Avramopoulos

**Affiliations:** Photonics Communications Research Laboratory, National Technical University of Athens, 9 Iroon Polytechniou Street, Zografou, 15773 Athens, Greece

**Keywords:** optical fiber sensors (OFSs), integrated photonic sensors, smart manufacturing, Industry 4.0, cyber-physical systems (CPSs), industrial monitoring, structural health monitoring (SHM), distributed fiber-optic sensing (DFOS), fiber Bragg gratings (FBGs), Rayleigh, Raman, Brillouin sensing, photonic integrated circuits (PICs), plasmonic/nanophotonic sensors, pipes, energy infrastructure, process monitoring, harsh manufacturing, in situ spectroscopy, artificial intelligence (AI), digital signal processing (DSP), machine learning (ML), sensor fusion, digital twins (DTs)

## Abstract

**Highlights:**

**What are the main findings?**

- Optical sensing in industry and smart manufacturing is not a single platform choice. Distributed fiber sensing, FBG sensors, integrated photonic sensors, and nanophotonic/plasmonic devices address different combinations of spatial coverage, sensing volume, measurand type, and interrogation complexity.
- While fiber Bragg gratings (FBGs) and DFOS remain the most mature platforms for embedded structural health monitoring (SHM), the deployment of integrated photonics (PICs) is currently bottlenecked by packaging, fiber–chip coupling, and calibration drift.

**What are the implications of the main findings?**

- To interpret complex sensor outputs into actionable state estimates and predictive maintenance, machine learning (ML), digital signal processing (DSP), and multi-modal sensor fusion must become part of the sensor deployment.
- Future work should move from sensing demonstrations toward qualified monitoring systems, where the optical sensor, interrogator, package, data-processing layer, and calibration process are designed together, able to supply calibrated physical data to digital twins.

**Abstract:**

Smart manufacturing, Industry 4.0, and cyber-physical systems (CPSs) require sensing architectures capable of resolving both spatially distributed asset behavior and highly localized process states. This review examines optical fiber sensors (OFSs) and integrated photonic sensors for industrial monitoring through a deployment-oriented, multi-scale perspective. The discussion covers five major application regimes: continuous infrastructure surveillance, structural health monitoring (SHM) of load-bearing composites, dynamic condition monitoring of machinery, in situ observability in advanced manufacturing, and localized chemical or gas sensing. Extended fiber-optic networks, including distributed fiber-optic sensing (DFOS) based on Rayleigh, Raman, and Brillouin scattering, together with multiplexed fiber Bragg grating (FBG) sensors, provide passive, embeddable, and remotely interrogated monitoring for large-scale assets and harsh environments. Photonic integrated circuits (PICs) shift transduction to compact node-level devices for localized thermal, mechanical, refractive-index, absorption, vibration, and inertial measurements, while plasmonic and dielectric nanophotonic sensors extend optical monitoring toward surface-selective and chemically specific detection. Across these platforms, digital signal processing (DSP), machine learning (ML), sensor fusion, and digital-twin (DT) coupling are treated as artificial-intelligence-enabled (AI-enabled) layers for signal recovery, inverse mapping, uncertainty reduction, and predictive maintenance. The review argues that scalable industrial adoption is less limited by sensing physics than by the complete deployment chain: packaging, fiber–chip interfacing, calibration stability, interrogation robustness, and AI-enabled data interpretation. This manuscript is structured as a deployment-oriented narrative review of optical fiber and integrated photonic sensors for industrial monitoring and smart manufacturing.

## 1. Introduction

The transition toward Industry 4.0 is reshaping industry and manufacturing around cyber-physical integration, real-time data acquisition, and autonomous decision-making [1]. Consequently, data-driven smart industry relies on sensing systems capable of continuous visibility into process states, equipment conditions, and product quality [2]. This requirement is inherently multi-scale. Manufacturing environments often demand monitoring of temperature, strain, vibration, pressure, displacement, and chemical or environmental state at very different spatial levels, ranging from localized component diagnostics to full-asset and process-line supervision. Therefore, the industrial sensing problem requires a delicate balance of sensitivity, coverage, spatial resolution, and field robustness.

Optical and photonic sensors address part of this deployment problem because they combine electromagnetic immunity, passive remote interrogation, multiplexing capability, and compatibility with harsh environments [3], where conventional electrical instrumentation fails due to noise susceptibility or wiring burdens [4]. Fiber-optic sensing enables both discrete and continuous monitoring over extended distances while remaining lightweight and embeddable [5]. However, no single optical or photonic technology covers every industrial scale, and thus, modern manufacturing demands a complementary and multi-scale sensing architecture, combining:

- **Fiber-optic sensors:** Defined by spatial continuity, these architectures dominate when extended reach, structural embeddability, and distributed coverage are required. They can be employed for high-resolution local mapping to extended-range asset monitoring [5,6].
- **Integrated photonic sensors:** Defined by localized, node-level transduction, these compact devices are packaged directly at the point of interest to provide optimal, embedded measurements compatible with chip-scale integration [7].
- **Plasmonic and nanophotonic sensors:** Defined by strong, surface-confined light–matter interactions and minimal sensing volumes, these platforms excel at highly localized, chemically specific detection. Dielectric nanophotonics achieve this extreme confinement without the inherent penalties of metallic plasmonic loss [8,9].

The review literature is well developed within each platform class, ranging from optical fiber sensing [10], distributed fiber-optic sensing [5], silicon photonic sensing [7], to plasmonic and nanophotonic sensing. The latter are often treated separately through surface-plasmon, dielectric-metasurface, photonic-crystal, or interrogation-focused perspectives [8,9,11,12]. What remains less common is a unified review that compares these platforms in terms of smart-manufacturing deployment, sensing scale, dominant measurands, and system-level complementarity.

**Figure 1:**
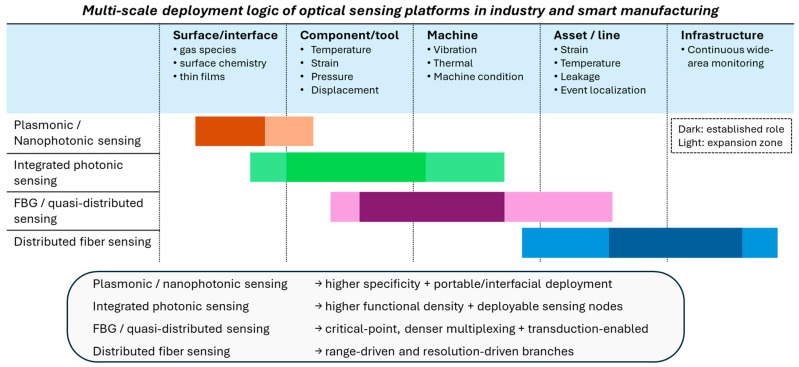
Multi-scale deployment logic of optical sensing platforms in smart industry and manufacturing. Optical sensing spans industrial measurement regimes from localized interfacial diagnostics to infrastructure-scale continuous monitoring. Dark bands denote established industrial roles, whereas lighter extensions indicate current directions of technological expansion.

In this manuscript, we treat industrial optical sensing as a multi-scale systems problem, shown in Figure 1. We compare fiber-optic, integrated photonic, and nanophotonic sensors based on transduction principles, industrial fit, and interrogation burdens. Following the fundamental sensing framework established in Section 2, Section 3, Section 4 and Section 5 critically examine the major optical platform families. Section 6 evaluates their deployment across specific smart industry and smart manufacturing applications, while Section 7 analyzes the critical role of AI-enabled data interpretation. Finally, Section 8 outlines deployment challenges and future research directions.

The central thesis of this review is that optical sensing in industry and smart manufacturing should be selected according to the measurement task, not according to platform specifications alone. Optical fiber, integrated photonic, plasmonic, and nanophotonic sensors are therefore treated as complementary measurement architectures with different industrial roles. Distributed fiber sensing is most appropriate when the required information is spatially continuous or must be recovered across inaccessible assets, process lines, or large structures. FBG networks are suited to passive, multiplexed measurements at discrete high-value points such as bonded joints, composite laminates, vessels, tools, bearings, and power equipment. Integrated photonic sensors become relevant when the measurement must be compact, localized, and close to a tool surface, process interface, or embedded electronic module. Plasmonic and nanophotonic sensors are reserved for cases where the measurand is surface-confined, interfacial, or chemically specific, and where the added burden of nanofabrication, fouling control, and packaging is acceptable. This platform-to-application logic is used throughout the review to connect sensing architecture, transduction mechanisms, optical observables, interrogation burdens, and deployment readiness.

### Review Methodology and Literature Selection

This manuscript was designed as a deployment-oriented narrative review rather than as a systematic review or quantitative meta-analysis. PRISMA 2020 reporting elements were therefore adopted only where applicable, primarily to document literature identification, screening, eligibility assessment, and inclusion. The literature-selection process is summarized in the PRISMA-style flow diagram provided as Figure S1, while the completed PRISMA 2020 checklist is provided as Table S1 in the Supplementary Materials. No statistical synthesis, effect-size estimation, or formal risk-of-bias assessment was performed. This approach was used to make the literature-selection process transparent while preserving the review’s comparative focus on sensing architecture, measurand type, interrogation burden, packaging constraints, and industrial deployment fit.

Literature was identified through searches in MDPI, IEEE Xplore, ScienceDirect, Web of Science, Scopus, SpringerLink, Optica Publishing Group, and ACS Publications. These searches were supplemented by Google Scholar searches, citation tracking, and reference-list screening to identify additional relevant implementation studies and platform-level reviews. The main search window covered 2006–2026, while earlier foundational works were retained when required to describe established sensing principles, including fiber Bragg gratings, distributed scattering-based sensing, integrated photonic sensors, plasmonic sensing, nanophotonic sensing, and optical-interrogation methods. Search strings combined platform terms, measurand terms, and application terms, including combinations of “distributed fiber optic sensing”, “Rayleigh sensing”, “Brillouin sensing”, “Raman temperature sensing”, “fiber Bragg grating”, “integrated photonic sensor”, “silicon photonic sensor”, “plasmonic gas sensor”, “nanophotonic sensor”, “smart manufacturing”, “industrial monitoring”, “sensor fusion”, “structural health monitoring”, “process monitoring”, “digital twin”, and “machine learning optical sensing”.

Papers were included when they satisfied at least two of the following technical criteria: reporting a representative sensing architecture, providing sensor performance metrics, addressing industrially relevant measurands, demonstrating harsh-environment or embedded deployment, describing interrogation constraints, reporting packaging limitations, presenting field validation, or applying AI-enabled interpretation to optical sensor data. Earlier foundational works and platform-level reviews were retained when they were necessary to explain established sensing principles. Papers were excluded when they were outside the industrial or manufacturing scope, lacked measurable sensing performance, or focused exclusively on laboratory-only sensing without industrial relevance. The included studies were categorized according to optical platform, measurand, spatial regime, interrogation burden, deployment condition, application area, and data-interpretation role.

The initial search identified 650 records. After removal of 90 duplicates, 560 records were screened by title and abstract. After initial exclusion of 240 records, and 25 reports not retrieved, a total of 295 full-text records were assessed for eligibility, of which 86 were excluded because they lacked industrial relevance, measurable sensing performance, deployment information, or a non-duplicative technical contribution. The final review included 209 records, which were categorized by optical platform, measurand, spatial regime, interrogation burden, deployment condition, and data-interpretation role.

## 2. Fundamentals of Optical and Photonic Sensing

Optical and photonic sensing transduces mechanical, thermal, chemical, or structural perturbations into measurable changes in wavelength, phase, intensity, frequency, or spectral shape [3,13]. These principles unify fiber-optic, integrated photonic, and nanophotonic sensors, even though the three platforms differ substantially in interaction length, sensing volume, and interrogation architecture. However, raw sensitivity alone does not dictate industrial value. Practical deployment demands the stable, interpretable recovery of a measurand, requiring strict coordination among the transduction mechanism, the optical observable, the interrogation method, and the physical deployment architecture. By systematically separating the physical measurand from the optical observable, these platforms can be evaluated across their spatial and functional regimes.

### 2.1. Optical Transduction Mechanisms and Interrogation Observables

Optical sensing requires a distinction between the physical measurand and the optical observable. The measurands are the physical quantities being monitored, such as temperature, strain, displacement, vibration, pressure, refractive index, or chemical concentration. These quantities perturb the optical path, effective refractive index, absorption, scattering response, coupling condition, or resonant behavior of the sensing structure [3,13]. The same physical quantity can be transduced through entirely different physical routes depending on the sensor platform selected. The choice of optical observable directly dictates system robustness and interrogation complexity:

- **Wavelength:** Tracking spectral shifts, such as the Bragg wavelength of a grating or the resonance of a photonic cavity, provides robust immunity against source-power fluctuations and transmission loss [3]. It is preferred for stable, multiplexed readouts and environments where optical loss tolerance is critical.
- **Phase:** Phase-based sensing is central to interferometric configurations, including Mach–Zehnder, Michelson, Fabry–Perot, and Sagnac architectures. It offers extreme sensitivity for displacement, strain, and vibration mapping. By resolving the measurand perturbations on the optical path-length, they deliver high precision at the cost of demanding rigorous coherence control, environmental stability, and complex demodulation schemes [3].
- **Intensity:** Measuring optical power variations (via absorption, scattering loss, coupling changes, or shadowing) allows for simplified readouts using fixed-wavelength sources and common photodetectors [3,13]. The inherent trade-off is a high susceptibility to non-sensing losses, source instability, environmental fluctuation, and alignment drift, requiring careful referencing.
- **Frequency:** Observable for distributed fiber sensing. Frequency-based methods encode physical information in the spectral properties of scattered light, recovered via OTDR- or OFDR-type interrogation [5,6]. Specific implementations include Raman for distributed temperature sensing [14], Brillouin for temperature and strain mapping [15], and coherent Rayleigh for distributed acoustic, strain, and temperature measurements [5,16]. While these architectures turn the fiber itself into a continuous spatial sensor and enable continuous spatial monitoring, they enforce strict trade-offs among sensing range, spatial resolution, acquisition time, and post-processing requirements [15].

**Figure 2:**
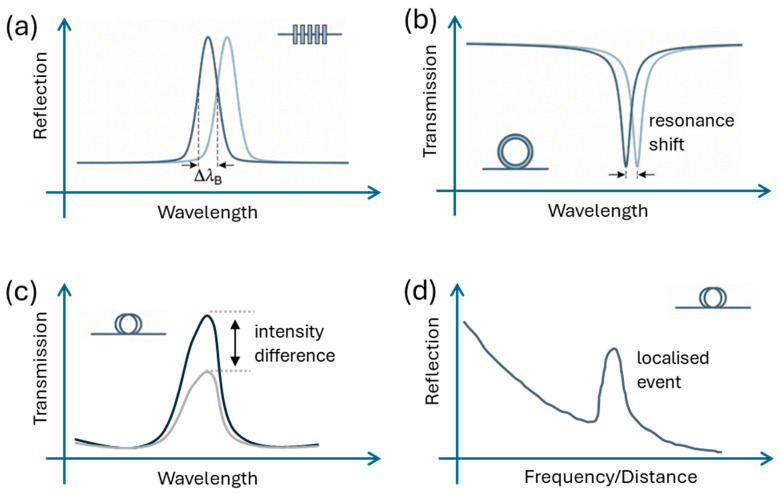
Optical transduction mechanisms and interrogation observables. A physical measurand perturbs the optical structure through a defined transduction mechanism, producing an observable change in: (**a**) wavelength, Inset: bragg grating, (**b**) phase, Inset: ring resonator, (**c**) intensity, Inset: fiber, and (**d**) frequency/distributed backscatter response, Inset: fiber.

The distinction among these observables is an engineering compromise. Wavelength readout dominates when stable feature tracking and massive multiplexing are the primary requirements. Phase readout excels for resolving sub-wavelength path-length perturbations, though it imposes a severe penalty in coherence control and demodulation complexity. Intensity-based readout suffices only where architectural simplicity outweighs the risk of uncompensated optical loss. Frequency- and backscatter-based interrogation is required when the sensing problem demands continuous spatial mapping, forcing the system to reconstruct information from distributed scattering along the entire optical path. The optical observables as well as the corresponding interrogation observables are summarized in Figure 2.

Industrial measurands, spanning temperature, strain, displacement, vibration, pressure, refractive index, and surface-bound interactions, cannot be captured by a single optical architecture. Platform selection must therefore begin with the nature of the measurand itself: whether it is localized or spatially extended, static or dynamic, directly observable or inferred through coupled optical effects. Optical architectures operate across distinct spatial measurement regimes:

- Fully Distributed Sensing: Fiber-based systems that continuously reconstruct spatial profiles along a measurement axis [5,6].
- Quasi-Distributed Sensing: Multiplexed point sensors (e.g., FBG arrays) that monitor discrete locations across a shared optical network [10,13].
- Localized Integrated Photonic Sensing: Compact waveguide circuits, resonators, or interferometers that realize transduction directly at a localized node [17].
- Highly Confined Plasmonic and Nanophotonic Sensing: Platforms that push confinement to the subwavelength regime, interrogating physical environments at specific surfaces or functionalized nanostructures [9].

The spatial regime must be distinguished from sensing locality and sensing volume. Because distributed fiber systems are widely used for extended assets, distributed sensing is frequently treated as synonymous with long-range monitoring. However, its defining feature is spatial continuity, and its practical implementations span vastly different operating regimes. Rayleigh- and OFDR-class methods deliver fine spatial discrimination over limited distances, whereas Raman- and Brillouin-based systems dominate extended-range thermal or thermomechanical monitoring [5]. Even when a high-resolution distributed technique reconstructs fine spatial variations, such as the 5 mm resolution achieved to monitor residual strain in additive manufacturing [18], the optical interaction itself remains extended along the entire fiber path, optimized for continuous mapping rather than local measurement.

Integrated photonic sensors occupy a fundamentally different regime, operating as truly localized transducers [7,17]. Their value lies in a reduced footprint and the strict confinement of the optical interaction to a bounded sensing region. This makes them the optimal choice when the measurand is strictly local, the required sensing volume is limited, or the transducer must couple closely with nearby instrumentation, electronics, or embedded packaging [19]. Plasmonic and nanophotonic platforms push this confinement even further, interrogating extremely small physical volumes [9]. While this extreme confinement delivers intrinsic chemical and interfacial specificity, it imposes practical limitations regarding susceptibility to optical loss, surface conditions, fabrication variations, and packaging.

Optical sensing enforces a strict system-level trade-off between sensitivity, stability, coverage, and interrogation burden. While distributed architectures yield continuous spatial data, they introduce constraints: demanding interrogation, heavy signal recovery and averaging strategy, dependence on stable optical power budgets, and environmental stability [5]. Extended-reach deployments escalate this burden further, requiring complex amplification, filtering, and dynamic-range management. Conversely, localized photonic and nanophotonic devices shift the balance in a different direction: they can deliver precise local response at the cost of tight packaging tolerances, careful stabilization, and demanding system integration requirements [20,21].

These platforms therefore operate as complementary, rather than competing, sensing architectures. Fiber-optic sensing is strongest when the monitoring objective requires spatial continuity, distributed reconstruction, or robust measurements over extended and difficult-to-access structures [5]. Integrated photonic sensing is utilized when the transduction is compact and localized [7,17]. Plasmonic and nanophotonic sensing is justified when the measurement depends on highly confined interaction with local thermal, mechanical, chemical, or interfacial perturbations [8,9]. Thus, the engineering mandate is not to declare a generically superior technology, but to deploy the exact physical architecture required by the specific industrial measurement problem.

**Figure 3:**
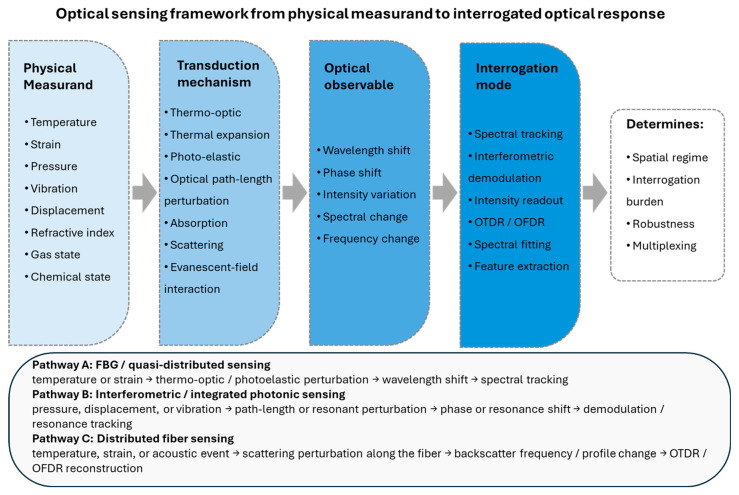
The optical transduction and interrogation chain. A physical measurand is converted into a sensor measurement through a sequence of physical perturbations, intermediate optical observables, and specific interrogation modes. This complete chain dictates the spatial regime, multiplexing capability, and ultimate industrial robustness of the sensing architecture. The indicative pathways illustrate representative implementations in FBG/quasi-distributed, interferometric/integrated photonic, and distributed fiber sensing.

The transition from a localized physical perturbation to a sensor measurement is governed by the complete transduction chain, as illustrated in Figure 3. This framework demonstrates that an optical observable is an intermediate state, not a final measurement. The chosen interrogation strategy enforces a system-level compromise: it dictates not only the spatial monitoring regime but also the multiplexing capability, the required signal-processing burden, and the robustness of the sensor in industrial environments. As a result, evaluating an optical architecture for industrial deployment requires assessing this entire chain.

### 2.2. Deployment-Level Comparison with Conventional Industrial Sensors

Optical sensing is best framed as a deployment-specific complement to established industrial sensors, with its value determined by the measurement geometry, operating environment, and interrogation constraints. Conventional mechanical, electrical, thermal, spectroscopic, and gas-sensing technologies remain preferable when the target variable is local, accessible, low-cost to measure, and compatible with wiring, surface contact, or line-of-sight access. Optical sensing becomes technically justified when the deployment imposes requirements for passive or remote interrogation, electromagnetic-interference immunity, dense multiplexing, spatially extended measurement, embedment inside structures or tooling, operation in high-voltage or harsh environments, or access to confined and hazardous regions [10,22]. Table 1 summarizes this deployment-level comparison by positioning representative conventional sensor families against the sensing conditions under which optical technologies become advantageous.

The comparison shows that optical sensing is not in general preferable for every industrial measurement. Conventional sensors remain the practical choice when the measurand is local, accessible, low-cost to monitor, and compatible with electrical wiring, surface contact, or line-of-sight observation. Optical sensing becomes more suitable when the deployment condition changes the measurement problem: when wiring density, electromagnetic interference, high voltage, distributed spatial coverage, embedment, hazardous access, or harsh-environment survivability becomes the dominant constraint. The relevant selection criterion is therefore not nominal sensitivity alone, but whether the sensing architecture can recover the required physical or chemical information under the installation, interrogation, calibration, and maintenance limits imposed by the industrial process.

## 3. Fiber-Optic Sensors

Optical fiber sensing remains the most deployable platform in industry, unifying remote interrogation, electromagnetic immunity, passive operation, corrosion resistance, and flexible routing within a single sensing medium [3,10,13]. In industrial environments, fiber optics are necessary when the sensing problem demands embeddability, passive operation in electrically noisy conditions, or measurements across geometries inaccessible to conventional electrical sensors. This versatility enables a broad spectrum of monitoring architectures, spanning from discrete embedded nodes to fully distributed spatial profiles across extended assets, components, and process lines [3,10]. Since platform selection is guided by the combination of measurand, spatial sensing mode, and interrogation burden, fiber Bragg grating (FBG) sensors are well suited for localized or quasi-distributed high-value points, whereas distributed scattering-based systems dominate when the sensing problem is spatially continuous [29].

### 3.1. Fiber Bragg Grating Sensors

Fiber Bragg gratings (FBGs) remain the preferred choice for localized industrial monitoring, offering compactness, passive operation, robust wavelength-division multiplexing (WDM), and relatively mature interrogation methods [30]. An FBG is formed by writing a periodic refractive-index modulation into the fiber core, creating a narrowband reflector within the fiber [31]. Its operation follows the Bragg condition,

$$\lambda_{B}=2n_{e}ff\Lambda$$

where $n_{e}ff$ is the effective refractive index and Λ is the grating period [30]. Any perturbation to the effective refractive index ($n_{e}ff$) or grating period (Λ) shifts the reflected wavelength to provide the sensing signal [31]. By fabricating individual gratings at distinct wavelengths, multiple sensing nodes can share a single optical fiber, enabling dense, quasi-distributed networks interrogated via standard WDM instrumentation [30]. This places FBGs between conventional point sensors and fully distributed systems, where the measurement remains discrete, but the sensors can still be deployed as dense quasi-distributed arrays [32].

Conventional FBG sensing is built around two measurands, axial strain and temperature [31]. Strain changes the grating period and modifies the effective refractive index through the photo-elastic effect, whereas temperature acts through thermal expansion and the thermo-optic response [33]. This direct wavelength encoding of mechanically and thermally relevant quantities is one of the main reasons FBGs became so useful in structural and manufacturing monitoring. However, it creates the limitation of strain and temperature being coupled in the grating response, so precise decoupling strategies are mandatory to isolate the variables [32]. Beyond strain and temperature measurements, FBGs continually evolve and expand their industrial deployment through novel architectures, functional materials, and harsh-environment implementations [22]. Other measurands, such as pressure, vibration, displacement, curvature, and acceleration, are interrogated by converting the target parameter into local strain at the grating element. Conversely, humidity and chemical measurands are recovered using specialized functional coatings that translate swelling, adsorption, or refractive-index shifts into measurable mechanical or optical perturbations of the grating region [32].

The practical value of FBG sensing is established by their ability to preserve stable wavelength transduction under thermal, mechanical, pressure, and chemically reactive environments. In embedded thermal monitoring in lithium-ion batteries, FBG arrays can resolve internal temperature differentials up to 8.6 °C under abnormal loads [29]. In strain-critical composite structures, a fully embedded FBG network for 70 MPa Type IV composite pressure vessels with 100% sensor survival have been demonstrated, with in situ monitoring beyond 172.4 MPa burst pressure, measured strains up to 18,000, and agreement within 10% of experiments and finite-element predictions [34]. The same FBG-based sensing platform can be extended through structural transduction, and develop pressure sensors for ship-bottom pressure-distribution monitoring with 58.94 pm/kPa sensitivity and 1.7 Pa precision, illustrating how local structural loading can be converted into accurate optical pressure readout at system scale deployment [35]. Chemical and gas sensing extends the platform further through functionalization. A representative hydrogen-sensing example is a side-polished FBG sensor, employing a WO3-Pd composite film and achieved hydrogen detection from 0.5 to 12,000 ppm with a response time of 0.9 s at 4000 ppm [36]. Recent work points to a more deployment-oriented stage, exploiting partially Pd-alloy-coated π-FBGs for linearized and temperature-compensated hydrogen sensing [37].

These examples define the strong position of FBG sensing in industry, a mature localized platform whose strengths remain strain and temperature, and whose wider impact comes from converting other industrial measurands into the same wavelength-encoded observable. This makes FBGs one of the most appropriate optical choices when the monitoring problem is concentrated at discrete high-value points and does not justify the full interrogation burden of distributed scatter-based sensing [38].

### 3.2. Distributed Fiber-Optic Sensing

Distributed fiber-optic sensing (DFOS) becomes the appropriate sensing architecture when the monitoring objective is a continuous spatial profile [6]. By interrogating scattered light, the optical fiber serves simultaneously as the sensing medium and the transmission path, reconstructing the physical response as a function of position [5]. This architecture is essential for localizing gradients, anomalies, or strain concentrations across spatially extended structures or process lines. DFOS is implemented via time-domain (OTDR) or frequency-domain (OFDR) schemes, enforcing an engineering balance between sensing range, spatial resolution, and measurement speed [5]. Consequently, the relevant technical distinction is how range, spatial continuity, measurand sensitivity, and interrogation burdens are optimized for the specific industrial measurement [5]. Practical implementations span three highly distinct operating principles:

- **Rayleigh Sensing:** Exploiting elastic scattering to track local optical path perturbations, Rayleigh systems support high-resolution strain and temperature mapping alongside distributed acoustic sensing (DAS) [5]. They provide fine spatial localization, making them highly effective for event-rich monitoring tasks such as intrusion detection, abnormal-event localization, and leak-related vibration tracking. For example, combining time- and frequency-domain features enables precise localization of abnormal events in underground pipelines [39]. OFDR-based Rayleigh sensing separates distributed sensing from long-range monitoring because its defining feature is spatially continuous interrogation, not sensing distance. This scale flexibility is evident in fiber-optic shape sensing, where distributed strain is converted into curvature and three-dimensional shape for medical instruments and flexible robotic structures [40]. In surgical-needle tracking, OFDR interrogation of high-backscatter fiber triplets reached 0.6 ± 0.2 mm precision with UV-exposed SMF-28 and a 1 cm gauge length [41]. A catheter-oriented implementation later reconstructed 20.6–40 cm shapes with mean errors of 0.8–2.6 mm and demonstrated real-time tracking at 30 Hz [42]. OFDR has also been extended to biochemical mapping over a 50 mm tapered-fiber sensing region with ultra-high sensing spatial resolution of 680 μm [43]. These examples position OFDR at the high-spatial-density end of distributed sensing, while Raman and Brillouin methods remain better suited to long-range thermal and thermomechanical coverage. However, high-resolution OFDR implementations impose short ranges and heavy interrogation requirements. Furthermore, calibration stability is dictated by coating-level environmental cross-sensitivities. While polymer-coated fibers exhibit a humidity-dependent decrease in temperature sensitivity, copper- and gold-coated variants provide robust, humidity-independent stability under identical conditions [44]. This is important for deployment in industrial environments because it shows that calibration stability depends not only on interrogation physics, but also on coating-mediated strain transfer and environmental robustness.
- **Raman Sensing:** Operating within a dedicated niche, Raman systems utilize the temperature dependence of Stokes and anti-Stokes scattering components for distributed temperature sensing (DTS) [14]. While they are not broad multi-measurand platforms like Rayleigh or FBG architectures, they are suited for thermal profiling where mechanical loading is not the primary concern, especially when monitoring long assets and extended industrial environments. Field deployments validate this capability, where pulse-coded Raman DTS has successfully reconstructed temperature profiles across 59 km of single-mode fiber with 2 m spatial resolution and 3.9 °C accuracy within 330 s [45].
- **Brillouin Sensing:** Because the Brillouin frequency shift depends on both strain and temperature, this architecture is the optimal solution for continuous thermomechanical monitoring over long distances [15]. It is best suited to extended-asset monitoring, such as pipelines, geostructures, and civil infrastructure, where broad spatial coverage supersedes the need for millimeter-level spatial resolution. In oil-pipeline monitoring, straight-line Brillouin optical time-domain reflectometry (BOTDR) deployments detect leaks at flow rates of 1.1 m^3^/h, while integrating plastic-film wrapping improves sensitivity by an order of magnitude to 0.1 m^3^/h [46]. The inherent trade-off is that Brillouin systems typically yield lower spatial resolution and enforce a more demanding interrogation burden than short-range Rayleigh configurations.

Each fiber-sensing class is defined by its distinct interrogation physics and system-level requirements. High-resolution distributed schemes, particularly OFDR-class Rayleigh implementations, depend heavily on stable frequency sweeps, correlation fidelity, and intensive signal processing. Conversely, extended-range Raman and Brillouin architectures are constrained primarily by optical power budgets, strict averaging requirements, and the necessity for complex amplification and filtering strategies [5,14,15]. The fiber sensing architectures are summarized in Table 2, providing details on the optical observables, measurands, spatial regime, range and interrogation requirements.

### 3.3. Interrogation Complexity and System-Level Considerations

The selection of a fiber-optic architecture is determined as much by interrogation complexity, data rates, and signal-processing burdens as by underlying sensing physics [13]. The practical distinction between FBG and distributed architectures therefore lies in how each architecture loads the readout system during deployment.

FBG systems benefit from highly established wavelength-demodulation hardware and multiplexing strategies, making them the optimal solution when the monitoring objective is localized and does not justify the computational load of continuous scatter readout [32]. Their interrogation is more manageable than continuous backscatter readout, especially when the number of sensing points is moderate and the target measurands are localized strain or temperature [38]. However, system performance remains dependent on interrogation design. For example, high-speed wavelength-to-time mapping architectures achieve interrogation rates up to 264 MHz with wavelength errors below 20 pm, while remaining fully compatible with quasi-distributed TDM/WDM networks [47]. This demonstrates that while FBG readout is highly developed, its operational limits are set directly by the speed and precision of the hardware used.

Conversely, distributed sensing systems employ complex interrogation units, requiring the continuous recovery of the sensing response as a function of position [5,6]. Time- and frequency-domain backscatter analyses demand higher acquisition performance, complex demodulation, and intensive signal processing compared to discrete grating readouts [6]. Indicatively, achieving sensing range greater than 30 km, with 1 m spatial resolution and 1 K temperature uncertainty within 1 to 60 s, requires uncompromising optimization of the optical signal-to-noise ratio [48]. Even within distributed sensing, however, the limitations are not uniform. High-resolution schemes, particularly OFDR implementations, depend on stable frequency sweeps, correlation fidelity, and careful signal processing, whereas extended-range Raman- and Brillouin-based systems are more strongly constrained by optical power budget, averaging requirements, and, in many cases, amplification and filtering strategy. Distributed sensing is therefore preferred when the necessity for continuous spatial coverage outweighs this additional hardware and computational requirements. Table 3 shows representative implementations for fiber-optic sensing in industry monitoring, illustrating the practical capability of the different fiber-optic sensing architectures.

From a system-level perspective, fiber-optic sensing is best understood as a family of architectures whose industrial suitability depends on the combined requirements of measurand, spatial coverage, and interrogation complexity. When the problem is localized strain or temperature at critical points, FBGs are often the practical choice [32]. When the requirement is distributed temperature profiling over long assets, Raman systems are preferred [14]. When continuous distributed strain and temperature monitoring over infrastructure-scale geometries is required, Brillouin methods become more defensible despite the heavier readout implementation [15]. In the case of distributed vibration, acoustic activity, or spatially localized abnormal events, Rayleigh-based distributed sensing becomes especially attractive [5]. The relevant comparison is therefore application-specific: the selected fiber-optic architecture must deliver the required spatial and measurand information while keeping readout complexity, processing load, and interpretation uncertainty within practical limits for the target industrial application.

## 4. Integrated Photonic Sensors

Integrated photonic sensing transitions optical measurement from extended fiber networks to lithographically defined, chip-scale circuits, prioritizing compactness, functional density, integration, and embedment. Photonic integrated circuits (PICs) are suited when measurements must be localized and executed directly at the point of interest. In industrial environments, this suggests their deployment for embedded diagnostics, in situ process monitoring, compact safety nodes, and applications requiring coexistence with electronics inside space-constrained or electromagnetically noisy equipment [49,50]. This localized role is not a concession to spatial resolution. While Rayleigh and OFDR fiber architectures can resolve fine spatial variations, they operate within a continuous mapping paradigm. Integrated photonics occupy a fundamentally different physical regime: they execute transduction directly at a compact node. By leveraging lithographically defined optical paths and engineered resonance or interference structures, PICs achieve direct compatibility with packaging and embedded deployment. Their industrial value therefore lies in node-level implementation and compactness, multi-functional sensing, and the ability to place the optical transducer at the point where the relevant thermal, mechanical, chemical, or process variable must be monitored. However, these advantages are accompanied by substantial system-level constraints. Because the sensing range is local, practical deployment remains constrained by susceptibility to thermal drift, fabrication variations, rigorous packaging requirements, alignment-sensitive fiber–chip interfaces, and the complexities of electronic co-integration [17,49,50]. PIC sensors should therefore be positioned as complements to fiber sensors, suited to measurements requiring compact node-level transduction, dense on-chip functionality, and close integration with local electronics or packaging.

### 4.1. Photonic Integration Platforms

The sensing behavior of a PIC is strongly shaped by its material platform. Optical confinement, propagation loss, device footprint, spectral range, and thermal drift differ substantially between silicon, silicon nitride, indium phosphide, and polymer photonics. These differences determine which platform is realistic for a given industrial sensor, because sensitivity must be balanced against manufacturability, environmental stability, packaging tolerance, and electronic integration [17,49,50,51,52]. The main photonic platforms are:

- **Silicon Photonics:** The main integrated photonics platform, leveraging high index contrast and CMOS-compatible fabrication for dense integration with access to a mature foundry ecosystem [49,50]. It is the optimal choice for compact resonant and interferometric devices where multiplexing density and electronic co-integration are of high importance. However, its strong thermo-optic response, sensitivity to process variation, and comparatively high propagation loss induce constraints on calibration stability and spectral drift in fluctuating environments [17,49].
- **Silicon Nitride:** Provides low propagation loss, a broad transparency window, and superior high-Q operation. It is well suited to integrated sensors where low loss, narrow linewidths, spectral stability, and low-noise readouts are prioritized over the very high compactness enabled by high-index silicon [17,52].
- **Indium Phosphide (InP):** Essential when native active functionality is required, supporting the monolithic integration of lasers, amplifiers, modulators, and detectors directly on-chip [51].
- **Polymer Photonics:** Prioritizes mechanical compliance, tunable thermo-mechanical response, and format flexibility over absolute integration density. It excels when the sensor must conform to bounded surfaces, operate as a lightweight embedded layer, or provide multi-axial information. For example, imprinted thin-polymer Bragg-grating foils multiplexed within a single waveguide achieve planar strain and temperature sensitivities of 0.85 pm/με and −150 pm/°C, respectively [53]. Despite these capabilities, polymer platforms currently lack the long-term thermal stability, robust packaging, and foundry-scale standardization inherent to silicon-based technologies, which limits their industrial adoption.

The main PIC platforms are summarized in Table 4, clarifying how platform choice affects confinement, propagation loss, spectral stability, active-functionality access, packaging tolerance, and industrial sensing fit. In practice, silicon is favored for dense integration and scalable fabrication, SiN for lower-loss and spectrally stable sensing, polymer photonics for compliant or surface-conformal formats, and InP when active photonic functionality is required. This comparison is platform-level, and the industrial value of a PIC sensor is ultimately set by the combined choice of material platform, transduction architecture, packaging method, and target measurand.

### 4.2. Sensing Quantities and On-Chip Transduction Architectures

Photonic integrated circuits (PICs) support a broad spectrum of industrial measurands, specified by how the optical interaction is engineered at the chip level. This quantity-based framing is useful, since the same device family can support very different sensing roles depending on how the optical interaction is engineered and what physical variable is being recovered. The optimal on-chip architectures are categorized by the physical variables they recover:

- **Refractive-index and absorption sensing** constitute the most established class of integrated photonic sensing for gas, fluid, and process-state monitoring [54]. By exploiting engineered evanescent-field interactions, compact resonators and interferometers convert refractive-index or absorption changes into measurable spectral or phase variations. For molecular absorption targets, waveguides and resonators function as miniaturized spectroscopic paths, seamlessly integrating sensing, wavelength discrimination, and readout directly onto a single chip. The industrial viability of this architecture is demonstrated through highly specific and compact implementations. Mid-infrared silicon waveguides achieve CO_2_ detection limits of 5000 ppm, proving that integrated absorption sensing operates effectively at the concentration levels required for workplace safety monitoring [55]. Silicon photonic dual-gas sensors enable the simultaneous detection of H_2_ and CO_2_, supporting localized industrial-safety and process-monitoring nodes [56]. When bulk sensing is insufficient, transduction is significantly enhanced by integrating chemically selective overlays. For instance, hybrid photonic cavities functionalized with metal–organic framework (MOF) coatings deliver highly specific detection of volatile organic compounds (VOCs) [57]. Similarly, silicon ring resonators utilizing ZIF-8/PDMS claddings enable the targeted sensing of dissolved CO_2_ [58]. These functionalization processes show that adding adsorption selectivity to standard guided-wave structures maximizes the capability of integrated photonics for localized chemical interrogation.
- **Temperature sensing** constitutes a highly mature integrated-photonic capability, driven by the strong thermo-optic response of waveguide structures that directly shifts resonances. Already early on it enabled scalable, chip-level thermometry, utilizing miniaturized silicon ring resonators fabricated directly in standard 0.18 μm CMOS processes [59]. Further extending this manufacturability, CMOS-compatible cladding-modulated silicon gratings achieve narrow-band reflection, high extinction ratio, and temperature sensitivity of 83.4 pm/°C across wide sensing ranges [60]. Furthermore, the industrial value of on-chip temperature sensing extends beyond standalone thermometry. It provides the essential thermal referencing required to stabilize other photonic sensors. Embedded temperature tracking enables direct, real-time drift compensation within complex structures, such as slot-waveguide ring-resonator refractive-index sensor arrays [61]. Similarly, integrated PTAT sensors provide the active thermal stabilization required for reliable micro-ring operation [62]. Temperature is therefore important in integrated photonics not only because it is easy to transduce, but because it acts as a reference for compensation and calibration purposes.
- **Strain, pressure, and displacement sensing** rely on mechanically compliant transduction structures to link optical signals with physical deformation. Consequently, interferometric, optomechanical, and membrane-coupled geometries form the foundation for these measurements. Pushing displacement sensitivity far beyond simple static wavelength shifts, integrated nano-optomechanical sensors achieve ultrawide optical bandwidths with imprecisions of just 45 fm/Hz^1/2^ and dynamic ranges exceeding 30 nm [63]. Pressure sensing is more mechanically design-dependent than thermometry, yet robust device-level implementations continue to mature. Integrated SiO_2_ arrayed waveguide grating (AWG) sensors, fabricated via standard planar lightwave circuit technology, provide highly linear measurements capable of withstanding pressures up to 40 MPa [64]. Similarly, silicon-on-insulator Mach–Zehnder architectures successfully translate membrane deformation into measurable on-chip optical signals. These devices prove that mechanical compliance must be engineered as an intrinsic component of the sensing architecture, rather than treated as a secondary packaging detail [65].
- **Vibration and acceleration sensing** in integrated photonics is based mainly on photonic MEMS, MOEMS, and cavity-optomechanical architectures, where inertial loading or mechanical displacement is converted into a measurable optical response. Packaging-aware implementations feature SOI MOEMS accelerometers embedded directly within metallic housings, achieving resonant frequencies of 1274 Hz and operating reliably up to 7 g [66]. Furthermore, integration density has advanced to support monolithically integrated, passive silicon tri-axial accelerometers operating over a 1–80 Hz band. These on-chip devices resolve average minimum detectable accelerations of 21.80, 24.77, and 32.47 ng/Hz^1/2^ across three axes, maintaining transverse crosstalk below 2.1% [67]. However, integrating these nano-opto-electro-mechanical devices alongside standard foundry components imposes system-level demands. Practical deployment requires rigorous wafer-level sealing, flip-chip bonding, and complex fiber-array attachment [68]. So, these devices provide the optimal architecture for localized dynamic diagnostics, where compact footprints and co-integration with moving elements are more important than broad spatial coverage.

Table 5 summarizes the main measurand classes addressed by integrated photonic sensing, linking each one to its dominant on-chip transduction architecture, representative quantitative evidence, and the primary deployment limitation that still governs industrial viability.

The comparison shows that integrated photonic sensing is not defined by a single device family, but by a set of localized transduction strategies whose maturity depends strongly on the measurand. Refractive-index and absorption sensing are the most established classes for localized process monitoring because they directly exploit evanescent-field interaction in waveguides and resonators. Temperature sensing is also mature, although its broader system-level role often lies in thermal referencing and drift compensation for more complex PIC sensors. By contrast, mechanically coupled quantities such as pressure, displacement, vibration, acceleration, and strain remain more dependent on transducer geometry, strain transfer, packaging stability, and optical interfacing.

### 4.3. Fabrication, Packaging, and Deployment Constraints

The practical industrial value of integrated photonic sensing is determined by manufacturability, reproducible packaging, and environmental stability, rather than nominal device sensitivity. Industrial deployment requires preserving the optical response long after fiber coupling, thermal cycling, electronic interfacing, and harsh environmental exposure are introduced [20,49,69].

- **Fabrication:** A major advantage of silicon and silicon-nitride platforms is their direct compatibility with semiconductor manufacturing, leveraging lithographic repeatability, wafer-level processing, and mature multi-project-wafer (MPW) foundry services [49,50,69,70]. However, scalable foundry access does not eliminate fundamental design trade-offs. High-Q resonators, narrow spectral features, suspended structures, and subwavelength geometries demand extreme lithographic control and suffer higher vulnerability to process variation [17,49]. Thus, raw theoretical sensitivity must be balanced against structural yield, reproducibility, and fabrication tolerances.
- **Packaging:** This remains the main bottleneck impeding scalable industrial adoption [19,20,60]. Fiber-to-chip interfaces, whether utilizing grating or edge couplers, demand sub-micron alignment and robust mechanical attachment, thermal dissipation, introducing optical loss, assembly complexity, and cost constraints [20,21,71]. Recent perspectives state that packaging remains a major barrier because cost and throughput still lag what is needed for broad deployment [21]. The field is responding through automation and assembly-oriented design, as illustrated by high-throughput photonic packaging work aimed at moving complexity away from bespoke alignment and toward scalable manufacturing workflows [71]. For example, microlens-enabled PIC packaging successfully relaxes alignment constraints, extends fiber–chip working distances, and supports the hybrid integration of external components, achieving a highly competitive 0.85 dB additional insertion loss per interface [72,73]. For optical sensors, however, packaging presents an additional challenge, where the enclosure must efficiently deliver light while simultaneously preserving the transducer’s physical access to the external environment, without degrading calibration stability. This translates device-level concepts into severe system-level packaging burdens. High-performance silicon photonic MEMS, for instance, mandate rigorous wafer-level sealing for long-term reliability, combined with flip-chip bonding and complex fiber-array attachments for optical and electrical interfacing, illustrating how quickly a sensor concept becomes a packaging problem at system level [68].
- **System integration:** Industrial deployment requires photonic sensors to be co-designed with electronic drivers, readout circuits, thermal management, calibration routines, and data-acquisition hardware, while maintaining coupling stability, drift control, parasitic-reflection suppression, and sufficient optical power budget [20,49]. These requirements are especially important in manufacturing environments, where compactness alone is not enough and the sensor must remain stable under vibration, temperature fluctuations, maintenance cycles, and prolonged operation. Integrated photonic sensors therefore remain limited less by sensing concept than by packaging, interfacing, and reliability at system level [20,68].

### 4.4. Industrial Positioning and Deployment Outlook

The industrial deployment of integrated photonic sensing is defined by compact, highly integrable nodes where conventional fiber routing is prohibitive. For this reason, the most relevant comparison is not with distributed fiber sensing, but with localized and quasi-distributed FBG architectures [32]. While FBGs provide robust, electromagnetically immune local measurements via remote interrogation, PICs elevate this capability by combining localized transduction, dense on-chip multiplexing, and intimate coupling with embedded electronics and readout hardware.

The industrial value of PIC sensing is therefore best defined by its functional role at the node level. Manufacturable CMOS platforms support true chip-scale thermometry [59], and advance from isolated circuits to fully packaged miniature thermal probes [74]. Simultaneously, integrated waveguides and resonators provide highly compact safety and process-state nodes, demonstrated by silicon-waveguide CO_2_ detection at 5000 ppm limits [55] and multi-functional silicon photonic chips simultaneously tracking H_2_ and CO_2_ [56]. Beyond primary sensing, PICs drastically reduce the footprint of the optical readout chain itself, where monolithic AWG-photodetector chips now interrogate external FBG arrays with 6.79 pm accuracy and 1 pm wavelength resolution [75], establishing the foundation for fully integrated, high-bandwidth on-chip interrogator modules [76].

However, the industrial promise of integrated photonics should not be overstated. The central barrier is no longer only device-level transduction, but the ability to deliver stable, packaged, field-ready systems with reliable fiber coupling, thermal control, calibration, and electronic interfacing [20]. Broader adoption remains severely constrained by the economic and engineering realities of automated high-throughput packaging, stable fiber coupling, and robust system integration rather than nominal device sensitivity [20,21,71]. So, integrated photonic sensing must be positioned as a specialized complement to fiber architectures. Fiber optics are suitable when the monitoring objective demands continuous spatial coverage, distributed reconstruction, or robust routing over extended domains. Conversely, PIC platforms provide the suitable architecture when compactness, localized interfacing, multiplexed on-chip functionality, and direct co-integration with control electronics constitute the decisive engineering factors. Near-term manufacturing deployment is most realistic for embedded thermal monitoring, compact gas and process-state sensors, chip-scale mechanical diagnostics, and miniaturized optical interrogator modules. Wider adoption will depend less on nominal sensitivity gains than on robust packaging, stable optical interfaces, calibration control, and reliable system integration.

## 5. Plasmonic and Nanophotonic Sensors

Plasmonic and nanophotonic sensing pushes optical confinement into the subwavelength regime, utilizing engineered nanostructures to maximize local light–matter interaction within minimal physical volumes. This extreme confinement differentiates these platforms from both extended fiber networks and standard integrated photonic circuits. While distributed fiber systems can achieve high spatial resolution, their optical interaction remains extended along the entire transmission path, optimized for continuous mapping. In contrast, plasmonic and nanophotonic sensors restrict transduction to bounded surfaces, interfaces, cavities, or functionalized nanostructures.

This confinement gives these sensors a specialized industrial role. These platforms are most appropriate when the target measurand is localized, interfacial, or chemically specific. However, maximizing near-field interaction introduces unavoidable system-level constraints. Operating at the subwavelength scale escalates device susceptibility to optical loss, dimensional fabrication errors, surface contamination, and packaging demands. As a result, within industrial and smart manufacturing environments, plasmonic and nanophotonic devices must be positioned as high-sensitivity extensions of localized integrated photonic sensors, deployed when the measurement demands very high specificity and sensitivity.

### 5.1. Plasmonic Sensing

Plasmonic sensing is governed by the excitation of surface plasmons at metal–dielectric interfaces, utilizing either propagating surface plasmon resonance (SPR) or localized surface plasmon resonance (LSPR) within metallic nanostructures [8,77]. They transduce refractive-index changes, surface adsorption, catalytic reactions, or molecular binding at the metal–dielectric interface into measurable spectral, angular, phase, or intensity changes within a subwavelength interaction volume [8,77].

The industrial credibility of plasmonics is established through its capability to interrogate highly localized chemical and gas measurands. Metasurface hydrogen sensors utilizing Pd nano-patchy particle arrays achieve response times below 3 s at 1 mbar H_2_, maintaining 2.5 ppm limits of detection (LOD) and full-scale accuracies exceeding 2.5% [78]. Overcoming the deployment barrier of humid operation, nanoplasmonic architectures demonstrate 100 ppm LODs in synthetic air at 80% relative humidity. These devices comply with ISO 26142:2010 [79] stability requirements down to 0.06% H_2_ and exhibit no measurable performance degradation after 140 h of continuous operation [80]. Further extending this environmental robustness, catalytic-plasmonic Pt nanoparticle sensors sustain stable responses over 143 h in humid air across 0–80% relative humidity, resolving 30–50 ppm H_2_ at temperatures of 100 °C and 80 °C, respectively [81]. Plasmonic transduction is therefore most useful in compact gas-safety nodes where hydrogen uptake, adsorption, or catalytic surface reactions at a functionalized metal interface must be converted into an optical signal.

However, their strong near-field enhancement is physically linked with metallic optical loss, spectral broadening, and susceptibility to drift driven by surface contamination and functional-layer aging. The transition from device-level sensitivity to routine industrial deployment remains bottlenecked by the economic and engineering demands of reproducible nanofabrication, substrate stability, and robust interfacial packaging [77,82]. Therefore, plasmonic devices must be positioned strictly as highly specialized transducers for gas safety, interfacial chemistry, trace contamination, or surface-reaction monitoring at localized critical points where optical fibers cannot be routed and standard integrated photonics cannot achieve the targeted sensitivity and specificity values [77,82].

### 5.2. Dielectric Nanophotonic Sensing

Dielectric nanophotonic sensing achieves high optical confinement without the constraints of metallic plasmons. By engineering optical resonances in photonic crystals, nanocavities, guided-mode-resonance structures, suspended nanowaveguides, and dielectric metasurfaces, these elements exhibit lower absorption loss, narrower resonances, higher quality factors, and reduced resonance-induced heating while maintaining direct surface access [83,84]. They emerge as a good solution when high spectral fidelity, repeatability, or imaging-based readouts are prioritized higher than the absolute near-field intensity generated by metallic structures [83,84].

Bridging the gap between dielectric and plasmonic elements, hybrid plasmo-photonic devices embed localized plasmonic enhancement directly within guided-wave photonic architectures. This integration strategically confines plasmonic amplification to specific functional layers while preserving the routing, interference, and multiplexing capabilities of the photonic circuit. Practical implementations validate this approach, where ultracompact bimodal plasmo-photonic refractive-index sensors are successfully integrated on SU-8 waveguide platforms [85], while plasmo-photonic interferometers utilize liquid dielectric loading for simultaneous temperature sensing and thermo-optic-coefficient extraction [86]. These architectures prove that high sensitivity can be achieved together with the incorporation of plasmonic field enhancement into photonic architectures that remain compact, functionally versatile, and close to practical integrated sensing solutions [85,86].

Purely dielectric nanophotonic sensors are most compelling when low-loss resonances and confined optical modes are needed to detect small refractive-index, absorption, or surface-bound changes. Suspended nanophotonic waveguides achieve CO_2_ detection limits down to 20 ppb while enabling isotope-specific measurements directly on-chip [87]. Moving to high-density node sensing, integrating four photonic crystals directly onto the tip of a four-core optical fiber enables simultaneous concentration and temperature tracking, achieving a temperature-estimation RMSE of 0.45 °C [88]. Furthermore, dielectric metasurfaces paired with hyperspectral imaging support the portable readout of ultrathin surface interactions, enabling label-free sensing below three molecules per μm^2^ and direct spectral retrieval from a single image without conventional spectrometers [89]. Although demonstrated in a bioanalytical context, the result is important more broadly because it shows that nanophotonic surfaces can support large-area, imaging-based, and potentially portable readout of ultrathin surface interactions [89]. Pushing beyond nominal detection limits, nanophotonic development increasingly prioritizes selectivity and environmental robustness. Guided-mode-resonance H_2_ sensors utilizing Al_2_O_3_/WO_3_/Pd nanostructures successfully achieve 40 ppm resolutions while employing principal-component analysis to isolate the target signal from humidity and temperature variations [90]. Although response times currently remain on the minute scale, these functionalized nanostructures retain full operational functionality after 6 months of deployment, underscoring a critical shift toward the long-term calibration stability demanded by industrial deployment [90].

### 5.3. Industrial Relevance and Challenges

Within industry and smart manufacturing sensing, plasmonic and nanophotonic sensors function as highly specialized, high-performance extensions of localized photonics. By leveraging very high local sensitivity, minimal sensing volumes, direct surface access, and chemical functionalization, they are justified when interrogating gas plumes near critical enclosures, catalytic interfaces, ultrathin coatings, localized contamination events, or process-zone reactions that are not accessible through bulk thermomechanical monitoring alone [84,87,88,89]. However, the immediate industrial viability of these platforms remains bottlenecked by the realities of the full deployment chain. Practical adoption is constrained by the demands of reproducible nanofabrication, sub-nanometer dimensional tolerances, nanogap and linewidth control, and contamination resistance [82,83,90]. Moreover, preserving calibration stability requires mitigating drift under humidity and thermal cycling, ensuring the long-term integrity of catalytic receptor layers, and developing packaging that maintains direct analyte access without degrading optical fidelity. However, plasmonic heating and ohmic loss remain fundamental physical liabilities inseparable from near-field enhancement [82], and these sensors should meet industrial standards for cost-effectiveness and reproducibility before scaling up.

Summarizing, plasmonic and nanophotonic platforms represent the high-confinement frontier of optical measurement, fulfilling a highly specific role within complementary, multi-scale monitoring architectures. As optical confinement scales downward, from fiber-optic networks providing continuous, extended spatial coverage, to integrated photonic circuits executing compact embedded diagnostics, plasmonic and nanophotonic devices serve as highly sensitive nodes dedicated to fundamentally chemical, interfacial, ultrathin, or surface-selective transduction [82,83,90].

## 6. Optical Sensors in Industry and Smart Manufacturing

**Figure 4:**
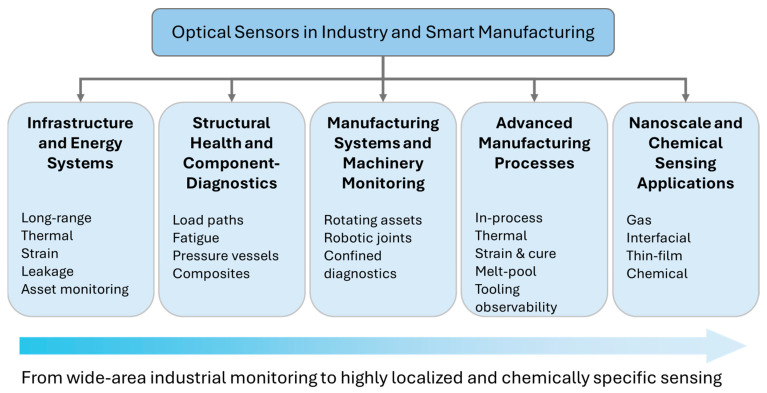
Application domains of optical sensors in smart industry and manufacturing.

The industrial relevance of optical sensing is specified by the specific application. In industrial applications, sensing challenges are defined by the intersection of spatial continuity, interaction locality, and deployment constraints, spanning from massive infrastructure and energy assets to engineered components, dynamic machinery, and chemically specific interfaces. Consequently, the selection of an optical platform depends mainly on whether the monitoring mandate requires wide-area distributed coverage, discrete critical-point interrogation, or strongly localized, surface-sensitive transduction. This application diversity states that optical sensing is inherently a multi-scale problem where fiber-optic, integrated photonic, and nanophotonic/plasmonic architectures serve as complementary layers. The following sections map these platforms across the industrial landscape and summarized in Figure 4, progressing from wide-scale infrastructure (Section 6.1) and structural health monitoring (Section 6.2) to dynamic machinery (Section 6.3), in-process advanced manufacturing (Section 6.4), and specialized nanoscale chemical diagnostics (Section 6.5).

### 6.1. Infrastructure and Energy Systems

Infrastructure and energy systems represent the largest monitoring scale in broad industrial environments, demanding reliable sensing across assets extending from hundreds of meters to tens of kilometers under severe weather exposure, mechanical loading, and electromagnetic interference. At this scale, the major requirement is not point sensitivity but continuous spatial coverage along pipelines, power cables, overhead transmission corridors, and other utility-scale assets. Distributed optical fiber sensing defines this application domain: Raman-, Brillouin-, and Rayleigh-based methods provide temperature, strain, and vibration information over long distances while retaining the durability and electromagnetic immunity needed in harsh operating environments [14,91].

**Figure 5:**
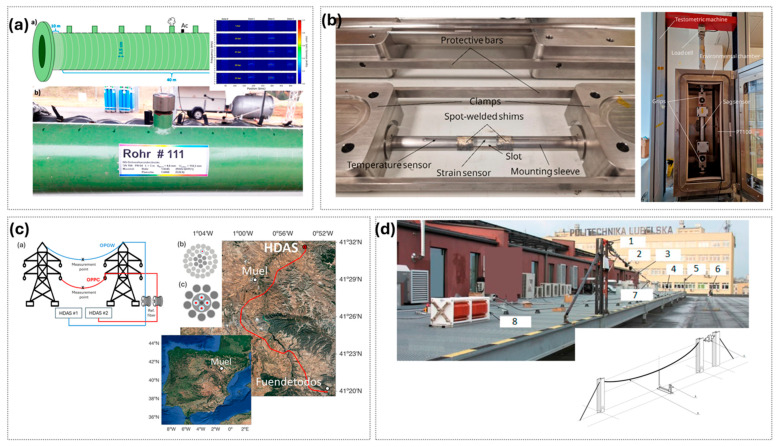
Representative fiber-based sensor implementations for infrastructure and energy systems. (**a**) a) Employed fiber application, b) Photo of instrumented pipe segment, Inset: Time-averaged DAS signal spectra [93], (**b**) (**Left**): Strain and temperature sensor pair welded to the mounting sleeve, (**Right**): Sag sensor setup [102], (**c**) (**Left**): Maps of overhead line installation (red line), and (a) Overhead installation scheme. (b) OPPC (fiber in red). (c) OPGW (fiber in red) [98], (**d**) Outdoor testbed with photonic sensor system embedded and its key elements. 1: Load cell; 2: proposed photonic sensor system; 3: temperature measurement; 4: sag measurement; 5: moving pole; 6: weather station; 7: ACSR Hawk conductor; 8: high-current transformer. Inset bottom: Outline of the outdoor testbed [100]. [All panels are adapted and reused under the terms of the Creative Commons Attribution 4.0 International License (CC BY 4.0)].

- **Pipeline and Utility-Corridor Monitoring:** In pipeline and utility-corridor monitoring, distributed fiber systems provide continuous thermal, strain, or acoustic information needed for leak detection, intrusion monitoring, and asset-state assessment. Field deployments integrate distributed temperature and acoustic sensing into SCADA networks for real-time LNG and LPG pipeline protection, spanning leak detection and physical intrusion monitoring [92]. For ultra-weak gas leaks, helical wrapping of the sensing fiber directly around the pipe amplifies distributed acoustic sensitivity, enabling the detection of leak-induced vibrations below 1%, as shown in Figure 5a [93]. For buried high-pressure gas lines, leakage-induced thermal anomalies are reliably detected when optical cables are positioned within 100 mm of the pipeline, whereas detection degrades rapidly at distances of 200 mm or beyond, while strategically deploying four cables within this 100 mm envelope successfully monitors the entire pipe cross-section [94]. Beyond raw acoustic and thermal physics, deployability is further enhanced by integrating optical fibers into structural textiles. For instance, BOTDR-based sensing textiles applied to HDPE pipelines accurately transduce strain behavior under 0 to 600 lb loads, proving that infrastructure-scale fiber sensing fundamentally relies on mechanical coupling to the host asset [95]. It also shows that infrastructure-scale fiber sensing is not only a question of interrogation physics, but also of how the sensing medium is physically integrated into the asset. These results highlight the comparative strength of distributed fiber sensing in infrastructure and energy systems: broad spatial coverage with useful localization over extended assets, while remaining compatible with practical installation strategies [92,93,94,95].
- **Electric-Power Infrastructure:** The core necessity is continuous thermal and mechanical monitoring over multi-kilometer conductors. Distributed temperature sensing deployed on XLPE-insulated 154 kV power cables achieves ±1 °C temperature resolution and 1.22 m spatial resolution under operational conditions [96]. Scaling to high-voltage underwater assets, online BOTDR systems embedded in 110 kV optical-fiber composite submarine cables map continuous thermomechanical profiles with 1 m spatial resolution [97]. For overhead transmission lines, chirped-pulse phase-sensitive OTDR interrogates both optical ground wires and phase conductors from a single endpoint covering up to 40 km, simultaneously tracking low-frequency thermal sag and high-frequency mechanical disturbances, as shown in Figure 5c [98]. These implementations show the infrastructure value of fiber-optic sensing, combining kilometer-scale reach with continuous thermal and mechanical monitoring.

Even within this distributed domain, specific high-value nodes, such as cable terminations, joints, and valve stations, demand dedicated point sensing. At these discrete hotspots FBG solutions have been demonstrated: FBG hoop-strain sensing for pipeline leakage and corrosion monitoring [99], FBG-based monitoring of overhead-transmission-line wire elongation and sag (Figure 5d) [100], and multiplexed FBG systems for multi-point cable-fault detection [101]. For pointwise conductor monitoring, a recent overhead-line FBG sensor using metal-coated gratings hermetically sealed in Kovar capillaries reported 0.4 nm/kN force sensitivity and 27 pm/°C temperature sensitivity over 30–200 °C (Figure 5b), while retaining force sensitivity within 20% over ten thermal cycles [102]. These results show that FBGs are relevant in this domain when the measurements can be reduced to localized variables such as strain, elongation, vibration, or fault-induced disturbance at selected positions. While integrated photonic sensors theoretically offer compact, high-density measurements for these critical nodes, their field deployment remains limited. Their practical use still depends on robust packaging, fiber-to-chip coupling, thermal stability, and environmental protection, all of which remain major barriers for field deployment [20,68]. To conclude, infrastructure and energy-system monitoring is a fiber-led domain, where distributed systems provide continuous wide-area coverage and FBG arrays secure localized hotspots, leaving integrated photonics as a future-state complement.

### 6.2. Structural Health Monitoring and Component-Level Diagnostics

**Figure 6:**
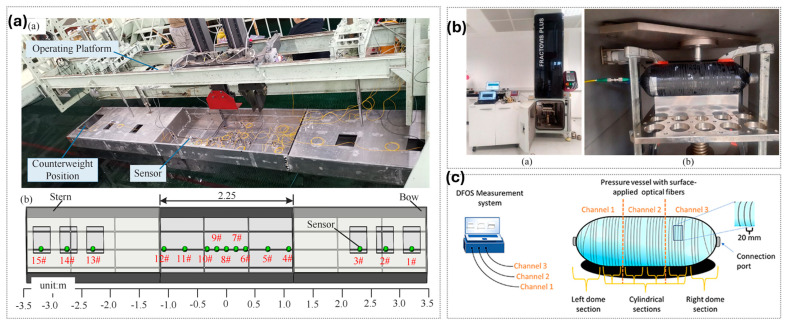
Representative optical−sensing implementations for SHM component diagnostics. (**a**) FBG pressure sensor layout on the ship. (a) Experimental test site, (b) Schematic diagram of sensor position [35], (**b**) (a) Dropweight impact testing setup with optical interrogation system, (b) composite overwrapped pressure vessel support [108], (**c**) Distributed fiber optic measurement system and the COPV with surface-applied optical fibers. The system uses three channels, each connected to an optical fiber monitoring area [110]. [All panels are adapted and reused under the terms of the Creative Commons Attribution 4.0 International License (CC BY 4.0)].

Structural and component-level monitoring addresses load-bearing assets where degradation develops through spatially non-uniform mechanisms, such as fatigue-driven strain redistribution, interfacial damage, and pressure-induced stress. Because these assets influence process reliability and operational safety from manufacture through service, optical sensing provides an electrically passive monitoring route for strain redistribution, temperature evolution, pressure loading, and damage-related structural changes. In practice, fiber-optic sensing, specifically distributed profiling and quasi-distributed FBG arrays, remains the most vital solution, scaling from embedded discrete points to continuous full-asset surveillance [1,103,104]. The industrial implementation of optical structural health monitoring (SHM) is defined across three distinct assets:

- **Composite and Laminated Structures:** In composite and laminated structures, damage often develops through interfacial debonding, delamination, matrix cracking, and local strain redistribution, making embeddable or surface-conformal optical sensing particularly valuable. Embedded FBG networks operate well beyond coupon-level feasibility, utilizing 7-sensor arrays to actively track impact damage and strain evolution across composite wing assemblies during periodic fatigue testing [105]. In secondary-bonded woven E-glass/vinyl ester joints, distributed embedded FBGs successfully track fatigue-driven crack growth by directly mapping local strain redistribution [106]. Operating survivability is validated through embedded FBG arrays deployed across fast patrol boats as shown in Figure 6a, covering spanning laminate, sandwich panel, and hull-level structures testing, yielding stable measurements under extreme dry-dock and sea-trial conditions [107]. These demonstrations show that, for composite SHM, the main value of optical sensing lies in survivable embedment and continuous strain-history capture from local interfaces up to full structural assemblies.
- **Bounded Shell-Like Structures:** In shell-like structures such as hulls, tanks, and curved panels, the monitoring task is often to map spatially varying pressure or strain over a bounded surface rather than along a single linear path. FBG arrays provide highly resolution monitoring across these extended geometries, successfully mapping ship-bottom pressure distributions utilizing networks of 15 sensors deployed across 6.75 m three-segment hull models, as shown in Figure 6a. These arrays operate with 58.94 pm/kPa sensitivity, 1.7 Pa precision, and 2.7 pm/°C temperature sensitivity, isolating complex structural responses during both static and dynamic wave-load interrogation [35].
- **Composite Overwrapped Pressure Vessels (COPVs) and Hydrogen Storage:** Safety-critical containment structures require thermomechanical monitoring because damage often initiates at winding transitions, bosses, and liner–composite interfaces. Embedded FBG networks can be integrated into automated fiber-placement workflows (Figure 6b), establishing continuous lifecycle visibility from fabrication to structural assessment, through 350 bar hydrostatic pressurization [108,109,110,111]. Pushing the operational limits, a fully embedded FBG network for 70 MPa Type IV hydrogen composite pressure vessels, reporting in situ monitoring above 172.4 MPa burst pressure, measured strains up to 18,000 με, 100% sensor survival, and agreement with experiments and FEM within 10% [34]. For retrofit SHM, enabling low-complexity installation after fabrication, surface-applied distributed fiber-optic sensing tracks dynamic hydrogen COPV pressure cycling between 20 and 875 bar at 5 cycles/min (Figure 6c), achieving 2.6 mm spatial resolution and utilizing measured-to-model strain residuals as direct damage indicators [110]. So, surface-applied architectures provide the optimal solution for retrofit-oriented SHM on existing vessels, whereas embedded sensing remains mandatory for visibility during the fabrication process. These structures demand less continuous geometric reach but impose a requirement for high-density optical interrogation across localized, high-risk transition zones.

Expanding beyond discrete pressure-vessel diagnostics, complementary DAS, DTS, and FBG architectures provide continuous, safety-critical monitoring across the entire hydrogen infrastructure ecosystem, encompassing bulk storage tanks, transport pipelines, and wide-area leak-detection networks [111]. Hydrogen infrastructure imposes strict sensing requirements because high-pressure containment, leak detection, and transport monitoring must be performed without introducing electrical ignition risks or dense wiring near hazardous regions. Optical fiber systems address these requirements by combining passive remote interrogation, electromagnetic immunity, and spatially scalable coverage across both pressure vessels and distributed pipeline networks [111].

Composite laminates are governed by local strain redistribution and interfacial damage, necessitating embedded point or quasi-distributed sensing [105,106,107]. Extended pipelines prioritize wide-area event localization, favoring distributed fiber architectures [35]. Containment vessels and tanks occupy a critical intermediate regime, demanding both the geometric reach to map overall load paths and the local sensitivity required to interrogate damage-initiating zones [34,109]. Fiber-optic architectures remain the foundation for structural monitoring because they span between embedded, surface-mounted, and distributed deployment configurations across all three regimes [103,104].

Beyond these fiber architectures, integrated photonic sensors provide a credible solution for localized, high-resolution, multifunctional structural diagnostics. Their operational value lies in compactness, high sensing density, and potential electronic co-integration at critical structural nodes where local information density is more important than meter-scale coverage. Demonstrating this manufacturing viability, silicon photonic sensors successfully embedded in composite tooling survive 7 bar and 165 °C, over 6h production cycles, achieving 78.4 pm/°C thermo-optic sensitivity with 0.9787 linearity [112]. At the device level, topological photonic-crystal ring resonators push this miniaturization further, targeting 24.34 nm/GPa pressure sensitivities within a 7.5 µm × 6.5 µm footprint [113]. However, the deployment of photonic integrated circuits in load-bearing environments remains constrained by packaging and interface stability. Reliable physical implementation requires wafer-level sealing, flip-chip bonding, and robust fiber-array attachment so that optical coupling and calibration are preserved under mechanical and thermal loading [20,68]. Overall, fiber-optic sensors remain the most mature and widely deployed optical route for structural monitoring, while integrated photonic sensors are better positioned as localized complements for critical points where compactness and high sensing density are required.

### 6.3. Manufacturing Systems and Machinery Monitoring

**Figure 7:**
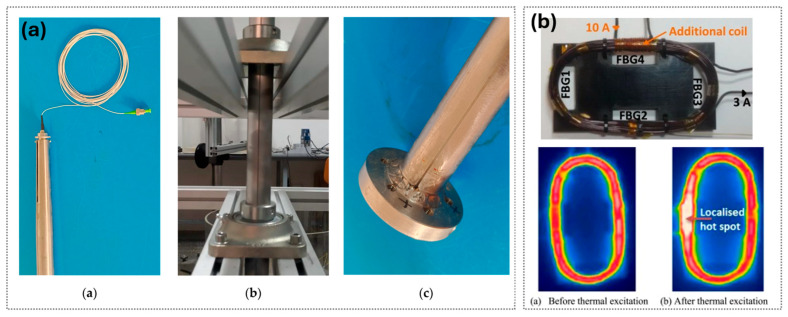
Representative optical-sensing implementations for manufacturing systems and machinery monitoring. (**a**) FBG sensors for monitoring shaft vibrations of hydraulic turbines. (a) Upper part of the shaft, (b) Groove machined along the shaft, (c) Bottom end of the shaft [115], (**b**) FBG sensor for electric coil monitoring. Top: Illustration of external coil use for localized high thermal stress, Bottom: Thermal images under non-uniform thermal stress, (a) Before, and (b) After thermal excitation [116]. [All panels are adapted and reused under the terms of the Creative Commons Attribution 4.0 International License (CC BY 4.0)].

Manufacturing systems and machinery monitoring encompasses dynamic machine assets whose immediate operational condition specifies production uptime, positioning accuracy, and maintenance scheduling. Unlike load-bearing structural assets, machinery diagnostics prioritize operating-state variables, such as transient vibration, localized strain, thermal buildup, and dynamic contact anomalies. Many of these systems include confined or electromagnetically noisy regions where conventional electrical probes are difficult to install and/or maintain, and gives optical sensing a clear practical role. Currently, FBG-based sensors resolve these dynamic variables [38], functioning across three primary automation machine categories:

- **Rotating Electromechanical Equipment:** For rotating assets, FBG-based sensing is the most appropriate diagnostic architecture because optical transducers can be positioned directly at the fault source, outperforming conventional housing-mounted vibration sensors. By placing FBG strain sensors in immediate proximity to the bearing, diagnostics successfully isolate both seeded and naturally developed faults across variable test rigs, drastically improving signal-to-noise ratios by eliminating remote vibration contamination [114]. This internal visibility extends seamlessly into the rotating components themselves, with 19-sensor FBG arrays embedded directly in active rotating shafts (Figure 7a), achieving 1 kHz interrogation rates within fundamentally confined kinematic structures [115]. Beyond mechanical vibration, multiplexed FBG networks provide real-time, distributed internal thermal monitoring directly within wound electric-machine coils (Figure 7b), resolving thermal states in geometries inaccessible to traditional instrumentation [116]. A complementary gearbox example has been reported, employing the tooth-root strain of a ring gear measured by FBG sensing to detect planetary gearbox tooth faults under both low- and high-speed conditions, thereby moving fault diagnosis closer to the mechanical source than standard acceleration-based methods [117]. Scaling to heavy manufacturing environments, FBG-based thermal-safety networks successfully interrogate backup bearings within the closed cavities of steel rolling mills [118]. The industrial value of optical machinery monitoring lies in the direct access to thermally and mechanically critical regions inside industrial process equipment, where conventional external instrumentation is often inadequate.
- **Robotic Manipulators and Automation:** Compact automation demands lightweight, mechanically compliant instrumentation capable of following continuous kinematic motion, detecting local contact, and operating within highly confined envelopes. Adapting to these requirements, discrete FBG arrays successfully map robot-joint microvibrations across a 50 Hz to 9 kHz operational band, indicating their suitability for dynamic measurements in compact robotic mechanisms [119]. Expanding from discrete joints to structural surfaces, multiplexed FBGs, embedded within smart textiles mounted directly on robotic manipulators, actively resolve the location, timing, and approximate force of external impacts, achieving measurement correlations exceeding R^2^ > 0.94 [120].
- **Heavy Machine-Tool Kinematics:** To maintain precise positioning accuracy during machining operations, FBG networks provide real-time observation of load-induced deformation across massive structural bases. In heavy-duty gantry machine tools in situ calibrated optical arrays successfully track these dynamic deformations, reducing maximum structural reconstruction errors from 69.53% down to 11% after in situ calibration [121].

Integrated photonic and optical MEMS platforms enter this domain strictly as localized inertial complements. Their engineering relevance lies in resolving multi-axis acceleration at highly confined nodes where absolute integration density supersedes broad machine coverage. Validating this compact format, packaging-aware SOI MOEMS accelerometers utilizing fiber-coupled readouts operate reliably up to 7 g with resonant frequencies of 1274 Hz [66]. Also, demonstrations show a monolithically integrated passive silicon tri-axial accelerometer for inertial operating over 1–80 Hz with minimum detectable accelerations of about 20–32 ng/Hz^1/2^, showing the performance now achievable in on-chip multi-axis inertial sensing [67]. However, exactly as observed in structural applications, deploying these chip-scale inertial sensors across real manufacturing hardware remains fundamentally bottlenecked by the complexities of robust packaging and optical interfacing [20]. Fiber-optic sensing therefore remains the mature optical route for machinery monitoring, whereas integrated photonic and optical MEMS devices are better positioned as emerging additions for localized, mainly inertial, diagnostics.

### 6.4. Advanced Manufacturing Processes

**Figure 8:**
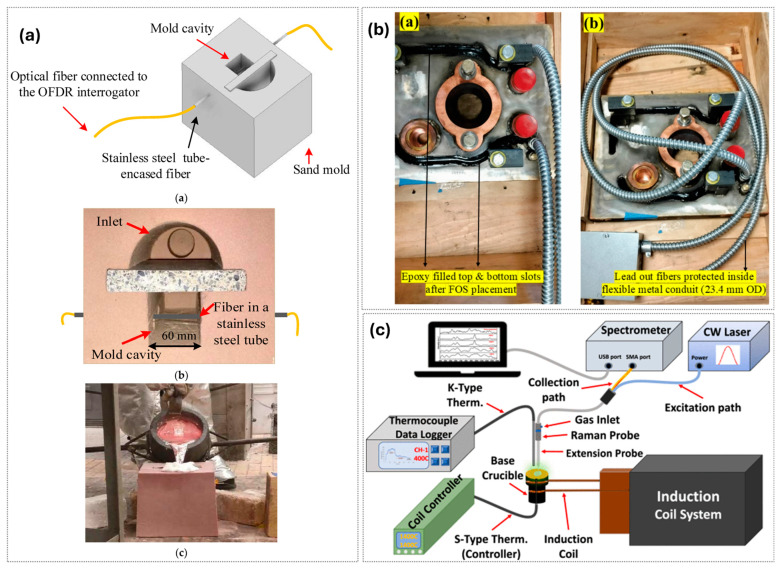
(**a**) Rayleigh-scattering-based fiber-optic sensor for monitoring of aluminum casting. (a) Experimental setup used for monitoring temperature across the mold cavity, during pouring aluminum and subsequent solidification, (b) Sand mold, illustrating the location of the fiber-optic temperature sensor, (c) Molten aluminum being poured into the mold [126], (**b**) Fiber optic sensors installed in the EAF copper injector panel. (a) Sensors secured in place with epoxy, (b) Fiber cables protected inside flexible metal conduit as they exit the panel [129], (**c**) Fiber-optic Raman system combined with an induction coil system for in situ high-temperature experiments [131]. [All panels are adapted and reused under the terms of the Creative Commons Attribution 4.0 International License (CC BY 4.0)].

**Figure 9:**
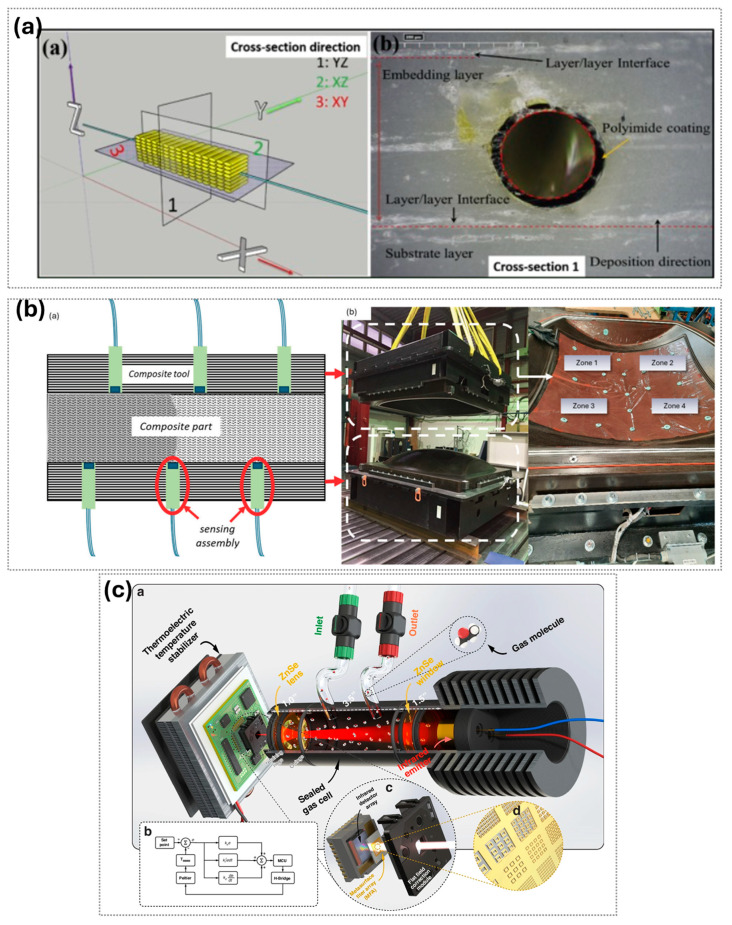
Representative optical-sensing implementations for advanced manufacturing and localized chemical/process monitoring. (**a**) Strain sensors in additive manufacturing specimens by using optical backscatter reflectometry. (a) Illustration of sampling position in specimen, (b) Optical micrograph of cross-section showing the SMF [124], (**b**) (a) Sensorized tool, with its deployed PIC sensor assembly, (b) Composite production tool, with the electrical wiring and optical fibers [112], (**c**) a. Schematic of metasurface gas sensing system. The MIMM and infrared emitter are placed on two sides of a custom-made gas cell. The MIMM is mounted on a thermoelectric temperature stabilizer. The gas cell is filled with gas to be sensed. b. Control loop diagram of the TTS controller. c. Schematic of the MIMM. Infrared radiation is spectrally filtered by a metasurface array, with transmitted light being absorbed by the pixels of the microbolometer camera. d. Schematic illustration of a metallic metasurface filter array [142]. [Figure 9a,c are adapted and reused under the terms of the Creative Commons Attribution 4.0 International License (CC BY 4.0), and Figure 9b is reproduced with permission from IEEE, copyright © 2025 IEEE, Giannis Poulopoulos].

Advanced manufacturing requires in-process visibility into transient thermal, mechanical, and chemical material states because these variables govern defect formation, residual stress, cure progression, melt behavior, process stability, and final part quality. Optical sensing provides an electrically passive route for measuring temperature, strain, resin flow, thermal fields, and reaction-state evolution within harsh or confined production environments. The most robust implementations transcend simple data logging, since they establish a direct physical link between in situ observables and resulting defects, residual stresses, distortion, cure state, molten-material behavior, or process chemistry [122,123,124]. This diagnostic logic extends beyond the workpiece to the production hardware itself, where tracking overheating, constrained-access failure risks, and machine states is essential for safe and stable process execution. An indicative demonstration shows FBG-based thermal monitoring of steel rolling mill bearings [118]. Representative implementations can be grouped into five manufacturing regimes:

- **High-Temperature and Molten-Material Process Monitoring:** High-temperature manufacturing is related to optical process monitoring in furnace hardware, casting molds, molten-metal interfaces, and steelmaking equipment. Fiber-optic high-temperature sensing has been reviewed for environments above 1000 °C, including metallurgy, fossil-fuel systems, aerospace, and power production, where small sensor size, electromagnetic-interference immunity, remote interrogation, multiplexing, and distributed measurement are the main deployment advantages [125]. In steel-industry-oriented Rayleigh-backscattering sensing, a stainless-steel-tube-encased optical fiber survived mechanically and optically above 700 °C during aluminum solidification in a sand mold in Figure 8a, while resolving spatial temperature features that conventional point measurements cannot capture [126]. In metal casting, a copper mold plate instrumented with a single continuous optical fiber generated thermal maps during cast-iron and steel dip tests in a 200 lb induction furnace, with maximum fiber-recorded temperatures of 469 °C and 388 °C, respectively, while the 0.65 mm, 25 Hz Rayleigh-OFDR measurements linked mold-temperature patterns to the solidified shell profile [127]. At furnace scale, Brillouin distributed temperature sensing was validated on the spray-cooled upper shell of a 150-ton direct-current electric arc furnace, resolving burner-activation and process-induced thermal events with 0.5–1 m spatial resolution and temperature trends consistent with resistance-temperature-detector readings [128]. A Rayleigh-OFDR deployment on an oxy-fuel burner/injector panel in a 77 metric ton direct-current electric arc furnace placed fibers 28.7 mm from the hot face (Figure 8b) and linked localized hot spots above 100 °C to furnace power, oxygen flow, carbon injection, and non-uniform slag coverage [129]. These implementations show that fiber sensing can convert high-temperature manufacturing hardware into a spatially resolved thermal diagnostic surface. However, high-temperature fiber sensing is not calibration-free: long-term tests of high-temperature-resistant FBGs at 900–1000 °C for more than 4000 h show that Bragg-wavelength drift affects the resonance used for temperature estimation, so pre-annealing, drift compensation, and calibration transfer remain necessary for long-duration furnace monitoring [130].
- **In Situ Spectroscopic Monitoring of Process Chemistry:** Several manufacturing processes require direct information on chemical state, molecular structure, or reaction evolution. In steelmaking, a portable fiber-optic Raman sensor was used for in situ mold-flux analysis at 1400 °C (Figure 8c), where Raman features in the alumina, silica, and silicate-network regions were correlated with flux chemistry, basicity, and viscosity-related indicators [131]. A later electric-arc-furnace slag study used a custom fiber-optic Raman probe to acquire spectra from molten synthetic slags at 1550 °C, resolving iron-related bands and silicate structural units. In that work, FeO_4_/FeO_6_ and silicate peak-area ratios were linked to Fe_2_O_3_ content, slag basicity, and silicate polymerization [27]. Beyond steelmaking, optical-fiber Raman spectroscopy has been reported for continuous monitoring of clinker and Portland-cement hydration during the first 8 h, identifying hydration products such as calcium silicate hydrate, ettringite, and monosulfate [132]. Fiber-optic sensors have also been applied to real-time curing-reaction monitoring in solid composite propellant, addressing the difficulty of observing physical and chemical cross-linking during propellant preparation [133]. These examples define a distinct role for optical sensing in manufacturing: direct in situ tracking of reaction state and process chemistry.
- **Additive Manufacturing:** In layer-by-layer fabrication, optical sensing bridges the gap between localized process signatures and structural integrity. High-speed infrared pyrometry and optical imaging deployed during laser powder bed fusion successfully capture conduction-to-keyhole transitions, enabling probabilistic predictions of pore formation [122]. Advancing beyond standard observation, single-camera two-wavelength imaging pyrometry integrated into commercial EOS M290 systems tracks melt-pool temperature evolution at 1 ms temporal resolutions and frame rates exceeding 30,000 fps across practical fatigue-specimen builds [123]. Complementing these surface measurements, distributed optical backscatter reflectometry embedded within material-extrusion processes shown in Figure 9a dynamically reveals internal residual-strain distributions directly as thermoplastic parts are printed [124]. Optical sensing in additive manufacturing transcends simple thermal logging, and functions as a critical diagnostic layer that maps transient process signatures directly to porosity, instability, and structural evolution.
- **Composite Curing and Consolidation:** Within composite manufacturing, the observables shift toward cure progression, resin flow, and residual strain. Embedded fiber Bragg gratings successfully in situ monitor cure-induced strain development directly within glass fiber/epoxy laminates during manufacture [134]. Validating this capability under rigorous autoclave conditions, in situ monitoring of [0_5_/90_5_]s laminates via embedded FBG arrays enables modified cure cycles that reduce thermal residual stress by approximately 50%, simultaneously increasing tensile strength to 1028 MPa and improving fatigue life by up to 614% [135]. In vacuum-assisted resin transfer molding (VaRTM), optical frequency domain reflectometry utilizing 100 mm long-gauge FBGs embedded in the 10th ply of a 20-layer, 400 mm × 100 mm glass-fabric preform successfully tracks vacuum compaction thickness reductions of up to 33%, while precisely localizing a deliberately introduced 23.7 mm unimpregnated defect through the distributed strain response [136]. Furthermore, in situ FBG networks actively discriminate between fabrication routes, quantifying how co-curing, co-bonding, and secondary bonding dictate room-temperature warpage in 150 mm × 150 mm autoclave-manufactured laminates [137]. In this context, optical sensors do more than record process conditions: they provide in situ evidence for comparing fabrication routes and quantifying how cure schedules and bonding strategies affect residual stress and geometric distortion.
- **Tool-Level Sensor Integration:** A critical evolution in this domain is the transition from monitoring the workpiece to embedding optical intelligence directly into the production tools. FBG arrays actively track tool-part interactions during the curing of AS4/8552 C-shaped composite structures on aluminum molds, linking tool-side measurements directly to spring-in distortion predictions [138]. Parallel advancements in metal additive manufacturing utilize distributed optical fibers embedded directly into printed metallic hosts to establish true in-process observability, despite the thermomechanical challenges of embedded deployment [139]. Pushing tool-level integration to its spatial limit, integrated photonics deliver highly localized, compact transduction directly inside production molds. Photonic integrated circuit temperature sensors are successfully deployed for out-of-autoclave composite production monitoring [140], while embedded phase-shifted Bragg grating sensors enable high-fidelity cure monitoring during resin-transfer molding [141]. In the most advanced implementations in Figure 9b, silicon-photonic sensors embedded within the production hardware itself track simultaneous thermal and cure evolution under realistic industrial processing conditions [112]. These advancements demonstrate the transition of photonic sensing from isolated laboratory-scale components into deployable manufacturing hardware capable of operating at the critical process interface [112].

Current demonstrations show a clear difference in deployment maturity across the optical sensing platforms. Fiber-optic sensing remains the established foundation for in-process monitoring because it can provide robust in situ measurements of temperature, strain, flow, cure evolution, distributed thermal fields, and process-state changes under realistic production conditions. Recent high-temperature and spectroscopic implementations extend this role from thermomechanical monitoring toward molten-material observability and in situ chemical-state tracking, particularly in steelmaking, casting, cement hydration, and reactive curing processes. While integrated photonics offer a compelling solution for highly localized, tool-embedded diagnostics, their viability remains bottlenecked by the physical realities of the deployment chain. Practical implementation in harsh manufacturing environments is constrained by alignment stability, thermal robustness, and the rigorous requirement for wafer-level sealing, flip-chip bonding, and standardized fiber interfacing [68,112], and thus integrated photonic sensors should be positioned as emerging, specialized complements for tool-level observability rather than as direct replacements of fiber-optic sensors.

### 6.5. Nanoscale and Chemical Sensing Applications

Nanoscale and chemical sensing become industrially interesting when the monitored variable is not bulk strain or temperature, but local gas composition, interfacial chemistry, or trace analytes confined to microscopic process regions. In these regimes, plasmonic and nanophotonic platforms are useful since they confine the optical field to subwavelength sensing volumes and strengthen light–matter interaction at the functionalized interface. Their industrial value resides in measurements that are chemically specific, surface-sensitive, and spatially localized, extending optical monitoring into the molecular domain where conventional thermomechanical fiber sensors are not designed to provide direct chemical or surface-selective sensitivity.

- **Gas-Safety and Process Analysis:** For localized chemical monitoring, implementation-driven platforms achieve laboratory-grade precision in industrial formats. Smart mid-infrared metasurface micro-spectrometers (shown in Figure 9c), utilizing filter arrays integrated with commercial cameras and machine learning, achieve 100% accuracy for CO_2_ and CH_4_ over 10–100% ranges within a footprint of 1 cm^3^ and 1 g. These platforms resolve hazardous gases with 98.4% accuracy, detecting NH_3_ at 100 ppm and methyl-ethyl-ketone at 200 ppm [142]. Hydrogen sensing provides a definitive case for industrial maturity, with nanoplasmonic sensors delivering 100 ppm detection in 80% relative humidity and maintaining full compliance with ISO 26142:2010 stability standards down to 0.06% H_2_ after 140 h of continuous operation [80]. Further overcoming the moisture barrier, catalytic-plasmonic Pt nanoparticle sensors resolve 30–50 ppm H_2_ concentrations over 143 h in humid air [81]. Pushing toward higher integration, on-chip plasmonic-catalytic nanojunctions operating at room temperature and zero bias achieve 1 ppm detection limits, achieving three orders of magnitude enhancement over non-plasmonic alternatives [143]. For high-selectivity applications, suspended nanophotonic waveguides now support isotope-specific CO_2_ sensing down to 20 ppb, addressing the most demanding requirements for process-gas and environmental analysis [87].
- **Interfacial and Thin-Film Diagnostics:** Nanophotonics enable the interrogation of atomic-layer-thick materials and surface-bound species where material volumes fundamentally elude bulk sensors. Pixelated dielectric metasurfaces featuring ultrasharp resonances support imaging-based molecular barcoding, providing chemically specific infrared readouts as spatially resolved maps [144]. These dielectric metasurfaces further support hyperspectral imaging and label-free detection below 3 molecules per μm^2^, while enabling the analysis of atomic-layer-thick materials over large areas [89]. For advanced manufacturing, this capability provides one viable route for monitoring thin functional coatings, adsorption layers, catalyst surfaces, and contamination films whose functional significance is high despite near-zero material volume.

The industrial role of these technologies is strictly specialized. Plasmonic and nanophotonic sensors are compelling when the measurand is inherently chemical, interfacial, or ultrathin, regimes where extreme local sensitivity justifies the significant overhead of nanofabrication and specialized packaging. These platforms serve as high-performance complements for gas-safety nodes and thin-film diagnostics at critical process interfaces.

Table 6 summarizes this application-domain logic by linking each sensing problem to the optical architecture, spatial role, and dominant deployment constraint discussed in the corresponding subsection.

**Figure 10:**
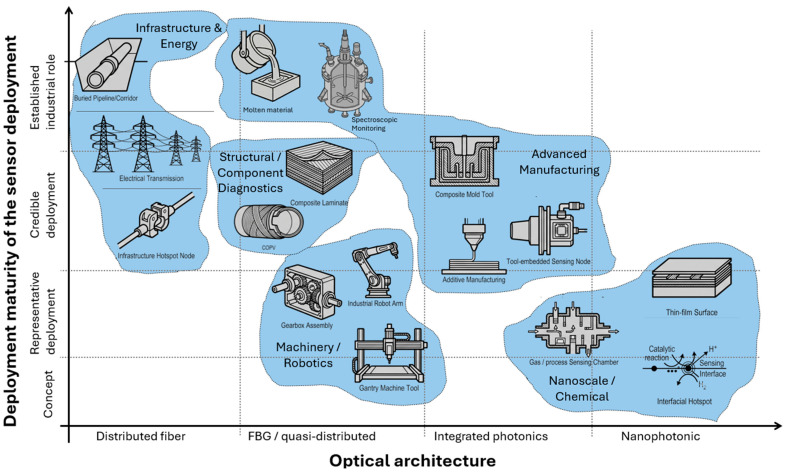
Application map of optical sensing in industry and smart manufacturing. Representative industrial sensing problems are positioned qualitatively according to the dominant optical-architecture regime and deployment maturity of the corresponding application–architecture pairing. Deployment maturity is assigned from the reviewed literature using field or near-field validation, interrogation maturity, packaging and installation robustness, calibration stability, and compatibility with industrial monitoring workflows.

The representative industrial and smart manufacturing sensing implementations show that optical sensing platforms do not progress toward industrial deployment along a single performance axis. Their suitability depends on the required spatial support, the physical coupling to the asset or process, and the maturity of packaging and interrogation. Figure 10 summarizes this application-level logic by positioning representative sensing problems according to optical architecture and deployment maturity. The map is qualitative rather than metric-based and its purpose is to show where each platform currently has the strongest deployment case. The horizontal position reflects the most suitable optical architecture required by the sensing problem, ranging from fully distributed fiber to quasi-distributed and chip-scale sensing. The vertical position reflects deployment maturity, assigned from the reviewed literature according to the following criteria:

- Field or near-field demonstration under industrial conditions
- Packaging, installation, and operational robustness
- Availability of mature interrogation hardware
- Calibration stability under environmental variation
- Compatibility with existing monitoring and maintenance workflows

Applications supported mainly by laboratory-scale demonstrations, custom packaging, or limited calibration evidence were placed at lower maturity, whereas applications with repeated field demonstrations, established interrogators, and clear installation practice were placed higher. The figure highlights two important deployment trends. First, infrastructure, structural, and machinery applications remain primarily fiber-led because they require spatial coverage, embeddability, or robust routing across industrial assets. Second, the transition toward tool-level process monitoring, gas-safety nodes, and interfacial diagnostics shifts the sensing requirement toward localized PIC and nanophotonic platforms, where compactness, chemical selectivity, and confined optical interaction become more relevant than long-range coverage.

## 7. Signal Processing, Artificial Intelligence, and Data Interpretation

Optical sensing platforms do not inherently output uniform physical measurements. Fiber gratings, interferometric devices, distributed backscatter systems, and localized photonic sensors generate distinct optical observables that include wavelength shifts, phase responses, spatially resolved scattering signatures, and compact spectral features. In smart industry, the primary engineering challenge is not simply acquiring these signals but converting them into reliable estimates of process states, structural integrity, or defect evolution, under the influence of varying environmental conditions, noise, drift, and cross-sensitivity. AI contributes along two distinct technical routes: first, by improving the sensing system itself through denoising, cross-sensitivity compensation, and signal processing, and second, by interpreting complex sensor outputs for classification, event detection, and failure forecasting [145]. Thus, when sensing architectures are localized around a specific interface, component, or process zone, data interpretation becomes part of the sensing architecture.

Digital signal processing (DSP) forms the first interpretation layer by denoising, demodulating, and stabilizing raw optical responses into usable spectral, phase, intensity, or time-domain observables before physical inference is attempted. Machine learning (ML) is subsequently deployed to decouple these signals when the relationship between the optical output and physical state is nonlinear, high-dimensional, or cross-sensitive. Multi-modal sensor fusion then integrates these estimates across different physical supports to resolve diagnostic ambiguity. Finally, at the system level, these interpreted optical streams feed digital twins, actively constraining process and structural models.

### 7.1. Digital Signal Processing for Enhanced Optical Sensing Performance

In optical sensing, the measured optical response has the role of an intermediate observable. Wavelength shifts, phase traces, resonance line shapes, and backscatter profiles do not constitute temperature, strain, or process-state estimates. Digital signal processing (DSP) operates directly within the sensing chain to recover, demodulate, denoise, and stabilize these responses before physical inference is possible [5,7]. In industrial monitoring, this processing stage determines how stable the reported measurand is and how quickly the system can update it. DSP is therefore not a secondary software layer; it stabilizes the raw outputs of distributed fiber, FBG, and integrated photonic sensors before they can be treated as deployable measurements.

- **Distributed Fiber Architectures:** DSP recovers usable spatial estimates from weak, multiplexed, or varying scatter signals. This requires phase demodulation, denoising, and the reconstruction of low-amplitude signatures that would otherwise be lost to attenuation or fading. Real-time phase demodulation over extended sensing ranges illustrates this constraint: signal recoverability depends not only on the fiber response, but also on the stability and latency of the processing chain [5,146].
- **Fiber Bragg Grating (FBG) Sensing Networks:** The processing focus shifts from spatial reconstruction to the stable extraction of Bragg-wavelength shifts from the rapidly acquired spectra. Techniques such as wavelength-to-time mapping combined with Gaussian filtering are implemented to preserve accurate shift estimation at high interrogation rates [47]. More recently, dual-optical-frequency-comb interrogation of fs-written FBGs with bandwidths of about 0.5 nm has shown that algorithm-specific spectral processing can materially improve low-strain performance, reaching 0.32 με resolution with approximately 0.8 pm fluctuation at constant strain, showcasing that FBG performance is increasingly constrained by the signal-recovery chain used to extract the Bragg shift [147]. System performance relies jointly on the optical architecture and the processing chain’s ability to maintain interpretability under high-sensing rate or low-SNR conditions.
- **Integrated Photonic Sensors:** The signal processing focuses on stable interpretation of compact resonant and interferometric readings. Measurement quality depends on tracking resonance wavelengths and linewidths under thermal drift, fabrication-induced variation, and source/read-out instability [7]. For silicon photonic ring-resonator sensors, active resonance-selection and tracking strategies are implemented to suppress line-shape distortion and preserve accuracy [148]. Phase-sensitive integrated sensors introduce additional complexities, requiring continuous phase recovery, drift compensation, and coordination with control loops [149]. As these circuits grow in complexity, sensing performance becomes more reliant on coordinated signal processing and feedback loops rather than photonic sensor performance alone [150]. As a result, the sensor performance is defined both by sensor sensitivity and by the signal-processing and control architecture used to recover and stabilize the readings.

These processing gains come with an implementation cost that directly affects deployability. In high-speed distributed sensing, FPGA-based demodulation has been used to sustain real-time extraction of weak signals [146]. Modular demodulation architectures have been implemented to maintain continuous operation under acquisition-rate and latency constraints [151]. Across optical sensing platforms, denoising, demodulation, and feature recovery are system-level assets only when they fit the timing, power, memory, and hardware limits of the interrogator or host electronics. So, DSP burden should be evaluated together with interrogation complexity. A sensing architecture is industrially credible only when the recovered optical observable can be delivered at the targeted update rate and latency without disproportionate hardware cost or control overhead.

### 7.2. Machine-Learning-Assisted Optical Sensing

Machine learning (ML) is useful in optical sensing when the measured spectrum, phase, intensity, or backscatter trace can no longer be mapped reliably to the measurand by a fixed calibration or closed-form inverse model. This situation arises when the optical output is high-dimensional, nonlinear with respect to the target state, cross-sensitive to multiple variables, or degraded by drift, baseline instability, and fabrication variability [152]. In these cases, ML serves classification, inverse mapping, parameter decoupling, and anomaly discrimination from optical observables whose relationship to the process state or structural condition is only partially known [152]. Recent implementations demonstrate that AI integration spans both localized configurations, including FBG, Fabry–Perot, and Mach–Zehnder sensors, as well as distributed Rayleigh-, Brillouin-, and Raman-based systems [145]. Within industrial environments, these data-driven approaches are categorized by the specific mathematical problem they resolve:

- **Spatiotemporal Event Classification:** In distributed fiber sensing, particularly DAS-type systems, ML is mainly established where large backscatter datasets must be assigned to specific physical events. The engineering difficulty is discriminating among weak, noisy, and overlapping readings associated with leakage, intrusion, impact, traffic, machinery activity, or damage-related disturbances along the sensing line [152]. Deep-learning-based multi-event recognition models are utilized to classify and localize these patterns at scales that are almost impractical for manual interpretation [153]. Convolutional, recurrent, and hybrid deep-learning models effectively convert these continuous backscatter measurements into event-level interpretations, although performance remains constrained by training-set coverage, class imbalance, and transferability across different environments or fiber installations [154]. Event-classification performance is governed by the full data pipeline, not only by the classifier architecture. A recent AI-driven DAS review identifies data acquisition, preprocessing, feature extraction, and model construction as distinct stages, while emphasizing the lack of public DAS datasets, high labeling cost, data-volume burden, and limited model generalization as persistent barriers [154]. This limitation is visible in microseismic DAS detection: a YOLOv3 model trained on synthetic events with real DAS noise addressed data volumes of about 650 GB/day for a 2 km cable sampled at 2000 Hz with 1 m channel spacing, detecting more than 80% of manually identified events in the main field dataset, but only more than 70% and more than 50% in previously unseen datasets with different acquisition geometries and recording conditions [155]. A separate ResNet50-based DAS study used 60,000 synthetic microseismic events and 10,000 field-noise records for simultaneous event detection, localization, and velocity-model inversion, explicitly noting that models trained in one situation require retraining or validation before transfer to different field conditions [156]. Industrial pipeline monitoring provides a complementary deployment case: a 48 km pipeline with 2400 observation points generated about 494 GB of real-site data, and a spatiotemporal recognition model achieved 99.26% accuracy at 500 Hz and 97.20% at 100 Hz with an 18.7 MB model suitable for embedded deployment [157]. Sensor-side data quality also affects classification reliability, as Rayleigh-enhanced DAS using femtosecond-laser-written scattering centers improved backscatter by more than 35 dB, reduced each acoustic event from 200,000 raw data points to 1280 preprocessed points, and achieved more than 76.25% supervised and 77.65% unsupervised recognition accuracy for human-locomotion events [158]. Active-learning results provide a route for reducing labeling burden under domain transfer, improving F1 score from 0.78 to 0.86 for transfer to a different well with the same sensor type and from 0.36 to 0.68 for transfer to a different well with different sensing equipment, while reducing labeling effort by more than 80% [159]. These examples indicate that DAS event classifiers should be validated across fiber routes, coupling states, acquisition systems, noise backgrounds, and deployment sites, not only through random splits of one installation-specific dataset.
- **Inverse Modeling and Parameter Decoupling:** When the mapping between optical response and physical state is nonlinear or cross-sensitive, data-driven inverse models are employed. In fiber-optic sensing, data-driven inverse models have been trained to estimate structural configuration directly from measured sensor outputs when analytical shape reconstruction loses flexibility [160]. A comparable inverse problem dictates FBG performance, where strain, temperature, and boundary conditions frequently generate overlapping spectral shifts. Machine-learning-assisted decoupling strategies recover these cross-sensitive quantities directly from FBG measurements, eliminating the reliance on additional calibration sensors [161,162]. This becomes relevant in manufacturing because the optical response often contains coupled thermal, mechanical, and process-dependent contributions that a single fixed calibration cannot separate reliably [161]. Representative Brillouin implementations show that inverse models must be evaluated across the coupled measurand space rather than only through random train/test splits. In BOFDA-based distributed humidity sensing, linear regression using Brillouin frequency shifts and linewidths decoupled relative humidity and temperature across 42 temperature–humidity combinations, covering 40–60 °C and 20–80%RH, with errors of 0.8 °C and 8.7%RH under leave-one-out validation [163]. Gaussian-process regression applied to four resolved Brillouin frequency-shift features discriminated temperature and strain over 20–40 °C and 0–1380 με, achieving 2 °C and 45 με errors and outperforming linear and ridge-regression baselines [164]. Brillouin-specific ML reviews further emphasize that validation on independent conditions, overfitting control, fixed input dimensions, interpretability, and uncertainty-aware prediction remain central barriers for deployment [165]. This inverse-mapping logic also extends to interacting damage signatures, where distributed fiber-optic strain profiles have been analyzed by machine learning to distinguish coincident cracks and corrosion with mAP@0.5 of 0.935, F1 score of 0.920, and analysis time below 0.009 s for profiles with more than 500 data points [166].
- **Multivariate Spectral Interpretation:** For compact photonic sensors, ML translates high-dimensional, multivariate outputs into physical measurements when responses are shaped by resonance distortion, fabrication variability, or baseline drift [167]. Data-driven analysis infers component composition within complex liquid mixtures directly from silicon-photonic spectral responses [168]. Pushing this integration further, compact mid-infrared metasurface micro-spectrometers combine a 1 cm^3^, 1 g photonic transducer with machine-learning-based identification to achieve 100% accuracy for CO_2_ and CH_4_ gas mixtures, while successfully detecting NH_3_ at 100 ppm [142]. However, the evidence base for these localized AI applications remains smaller than for DAS or FBG architectures. AI-assisted photonic noses remain difficult to deploy because baseline drift from contamination, aging, and changing ambient conditions requires repeated recalibration and field revalidation [169]. For photonic and spectroscopic classifiers, high accuracy on a controlled spectral library is not sufficient evidence of deployability. Model validation must include baseline drift, source/detector instability, coupling changes, chip-to-chip variability, surface contamination, humidity, and fouling. Otherwise, the classifier may learn sensor- or setup-specific spectral signatures rather than a transferable chemical or process state [145].

Despite these platform-specific capabilities, the industrial reliability of ML-assisted optical sensing is determined less by benchmark accuracy than by training-data representativeness, validation protocol, and stability of the sensor response under changing interrogation conditions. Recent AI OFS reviews identify scarce labeled datasets, calibration drift, sensor aging, environmental variability, domain shift, limited field validation, and limited integration with physics-based models as deployment barriers [145]. In DAS, these constraints are amplified by large data volumes, limited public datasets, high labeling cost, and weak generalization across different operating scenarios [154]. Industrial validation should test transfer across installations, process conditions, sensor batches, or acquisition systems, and should report confidence together with class labels or point estimates [165]. Active learning and few-shot learning can reduce labeling burden under domain transfer, but should still be accompanied by field validation under shifted sensor hardware [159], while physics-informed ML is relevant when the optical observable follows known physical constraints [170]. ML should therefore be treated as a constrained inverse-mapping layer that must remain calibrated, interpretable, and transferable across deployment environments.

### 7.3. Sensor Fusion and Multi-Modal Data Integration

Sensor fusion in optical manufacturing monitoring starts from the fact that different sensors rarely measure the same physical volume, time scale, or state variable. A distributed fiber system resolves a continuous profile along a structure or process line, an FBG array reports wavelength shifts at selected locations, and an integrated photonic sensor returns a localized spectral or interferometric response near an interface, or in a very confined area [171]. These channels are not interchangeable, even when they monitor the same process or structure, because each one samples a different physical volume, time scale, and optical observable. Fusion is credible only when the monitoring architecture preserves these differences. In practice, this is based on three engineering operations:

- **Data Alignment and Synchronization:** A local spectral response, a distributed strain profile, and a thermal or vision-based signature cannot be fused directly unless the monitoring system resolves spatial registration, temporal synchronization, preprocessing, normalization, and confidence weighting across channels with different sensing volumes [149]. In distributed architectures, these alignment operations must occur directly inside the monitoring pipeline to establish signal meaning through time and process context [172]. In additive manufacturing, this alignment challenge is successfully resolved by utilizing voxel-based data models, which align diverse sensor streams within a unified spatial framework prior to fusion [173].
- **Diagnostic Justification:** Multi-modal fusion is justified when process deviations or defects emerge across different physical signatures. Complementary channels add diagnostic value only when the fusion layer preserves their different sensitivities and physical roles [174]. In powder bed fusion, integrating layer-wise imagery, acoustic emissions, multispectral signals, and scan-vector information successfully correlates in situ observables with CT-detected flaws, significantly improving defect discrimination [175]. Furthermore, combining thermal and visual channels in laser-based powder bed fusion detects process abnormalities more selectively than either channel alone [176]. Similarly, in continuous-fiber composite additive manufacturing, integrating visual, infrared, force, and laser-displacement sensing enables the direct correlation of transient process signatures to layer-wise defects and final surface quality [177]. The value of feature-level fusion is also clear in pipeline monitoring, where synchronized DAS, FBG, and accelerometer measurements have been combined to couple distributed localization with pointwise strain calibration and high-frequency vibration capture, yielding a blockage-degree inversion model with R^2^ = 0.985 and mean relative prediction error of 3.77% [178]. Summarizing, fusion reduces state-estimation ambiguity that remains unresolved when each channel is interpreted on its own.
- **Deployment Barriers and Computational Overhead:** Despite its diagnostic power, multi-modal fusion imposes technical barriers. A distributed optical profile and a localized photonic response are not directly commensurate unless the fusion layer manages uncertainty propagation and synchronization [149]. In industrial environments, variable process conditions and real-time communication limits make this integration burden harder to control. For example, achieving real-time standoff prediction at 50 Hz during directed energy deposition requires the explicit, high-speed synchronization of 50 fps RGB camera imagery with 200 kSps acoustic data [179]. Recent work on LPBF sensor fusion concludes that while feature-level integration often provides the optimal balance between predictive performance and computational cost, limited ground truth, class imbalance, and the absence of standardization remain critical deployment obstacles [180]. So, modality selection becomes a foundational part of the fusion problem, since additional sensing channels are justified only when their distinct information gain outweighs the substantial synchronization, computational, and implementation overhead they introduce [181].

### 7.4. Digital Twins and Model-Integrated Industrial Monitoring

A digital twin in industry or manufacturing becomes valuable when measurement data updates a process or structural model enough to improve the estimation of the current system state. Optical sensing contributes by constraining thermal, mechanical, or process-state variables that raw measurements do not expose directly [182]. The key requirement is model updating derived from observables with sufficient spatial support, temporal fidelity, and calibration stability. The value of optical sensing is dictated entirely by how much the measured response reduces uncertainty in state estimation, defect evolution, or process interpretation under industrial operating conditions [183]. In additive manufacturing, digital twins actively connect in situ process measurements, model updating, and quality prediction across the build lifecycle [184]. Within this framework, the optical sensing layer functions across the following roles:

- **State-Constraining Observables and Model Validation:** Optical sensing provides spatially structured measurements that validate predicted evolution and test whether the twin remains physically consistent. In sensing-driven structural twins, distributed fiber-optic measurements are combined with inverse finite-element reconstruction to enable real-time deformation estimation in composite plates [185]. For inspection-oriented twins, distributed measurements integrate directly with BIM, augmented reality, and edge computing to support data-informed visualization [186]. This state-constraining role is critical in composites processing, where digital-twin frameworks couple with in-mold multi-sensor monitoring to track material flow and polymerization states [187]. Similarly, in intelligent assembly, optical-fiber networks operate as the real-state acquisition layer, enabling the online correction of assembly conditions [188]. For structural components, CFRP digital twins rely on these measurement layers for damage detection and state inference [189], while FBG arrays fused with finite-element models successfully reconstruct full-field temperature behavior to support leakage diagnosis [190].
- **Validation in Critical Assets:** The industrial credibility of these twins requires strict quantitative agreement between the predictive model and the physical sensor. In additive manufacturing, Rayleigh-OFDR interrogating optical fibers embedded in Inconel components during the LENS process actively monitors temperature and residual strain with 5 mm spatial resolution, successfully validating and refining the process twin [18]. For safety-critical hydrogen containment, surface-applied DFOS combined with model-predicted strain forms residual-based damage indicators during dynamic COPV pressure cycling between 20 and 875 bar at 5 cycles/min [110]. This directly supports the expansion of distributed and point optical sensors as safety-critical monitoring layers within hydrogen infrastructure [111]. Furthermore, DFOS and pressure data operate as the physical-data layer of a digital replica, enabling anomaly detection and remaining-lifetime prognosis in hydrogen pressure vessels [191].
- **Platform-Specific Twin Integration:** Optical sensing does not contribute uniform data to digital twins since the architecture must match the required model variables. Distributed fiber sensing is employed when the model requires spatially continuous field information, such as deformation, temperature, or strain evolution over an extended structure [185]. FBG arrays and discrete optical sensors are deployed when the twin is updated from selected high-value measurements at specific locations [190]. Compact integrated photonic or nanophotonic sensors become essential when the model depends on highly localized thermal, mechanical, chemical, or interfacial observables.
- **Deployment Constraints and Model–Sensor Coupling:** Digital-twin deployment is limited by practical system requirements: the sensor data must be coupled to a validated model, uncertainty must be tracked, and updates must remain computationally feasible under industrial operating conditions [192]. In optical monitoring, these integration constraints are amplified by calibration drift, inverse-reconstruction errors, synchronization demands, and the inherent difficulty of merging distributed profiles, discrete points, and localized photonic responses with fundamentally different spatial supports and uncertainty structures [182].

Industrial use of digital twins depends on whether the sensing architecture, model structure, and update workflow can deliver actionable state estimates within the time scale of the monitored process. Table 7 summarizes the role of data interpretation in optical monitoring by linking optical inputs to processing tasks, derived outputs, and industrial constraints.

**Figure 11:**
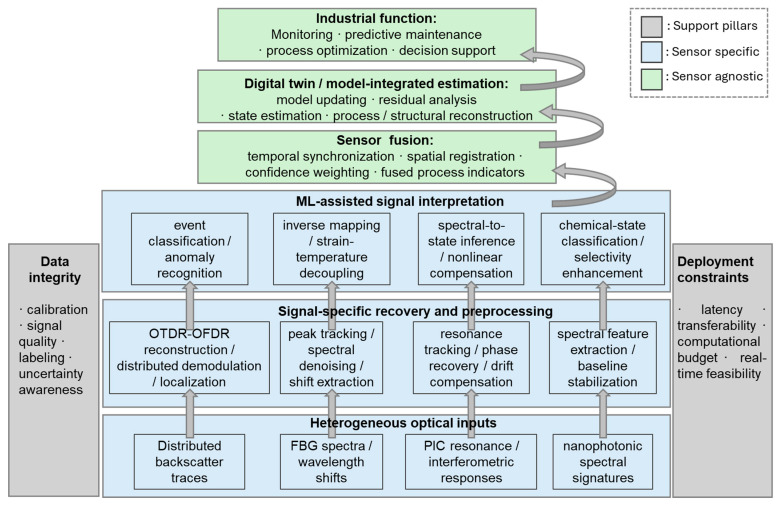
Interpretation hierarchy for optical sensing in manufacturing and industrial monitoring. Heterogeneous optical inputs from distributed fiber, FBG, integrated photonic, and nanophotonic sensors require signal-specific recovery before ML-assisted interpretation, sensor fusion, and digital-twin coupling can produce usable process or structural-state estimates. The diagram separates sensor-specific processing from sensor-agnostic decision layers.

Figure 11 summarizes the interpretation stack required to convert heterogeneous optical sensor outputs into industrially usable state information. Distributed backscatter traces, FBG wavelength shifts, PIC resonance or interferometric responses, and nanophotonic spectral signatures enter the system as optical observables. Signal-specific preprocessing first recovers stable features from each observable, including localization, peak tracking, phase recovery, drift compensation, and baseline stabilization. ML-assisted interpretation can then support inverse mapping, event classification, nonlinear compensation, or chemical-state classification, but only after the sensor-specific recovery step has preserved the physical meaning of the optical response. At higher levels, sensor fusion and digital-twin coupling combine these outputs into fused indicators, residuals, state estimates, or model-constrained reconstructions. The side pillars indicate that this computational chain remains deployable only when data integrity and implementation constraints, including calibration, uncertainty, latency, transferability, computational burden, and real-time feasibility are explicitly controlled.

## 8. Industrial Challenges and Implementation Barriers

In industrial environments, deployment is not limited by intrinsic sensitivity, but by interrogation complexity, packaging robustness, calibration stability, and the difficulty of integration into established process-monitoring workflows [2]. The bottleneck is platform specific: distributed fiber systems are limited mainly by interrogation burden, data volume, installation practice, and range/resolution/rate trade-offs. FBG systems are limited by multiplexing, and cross-sensitivity management; PICs by fiber–chip coupling, thermal drift, and package stability; and plasmonic/nanophotonic sensors by surface robustness, fouling, and reproducible nanofabrication. As a result, the critical challenge is the conversion of these solutions into reliable, scalable, and maintainable industrial systems that remain robust under the noise, drift, and non-stationary conditions of the industrial environment. These constraints accumulate across a deployment chain that begins with the sensing requirement itself and extends through sensor embedment, packaging, interrogation, signal recovery, and system integration.

**Figure 12:**
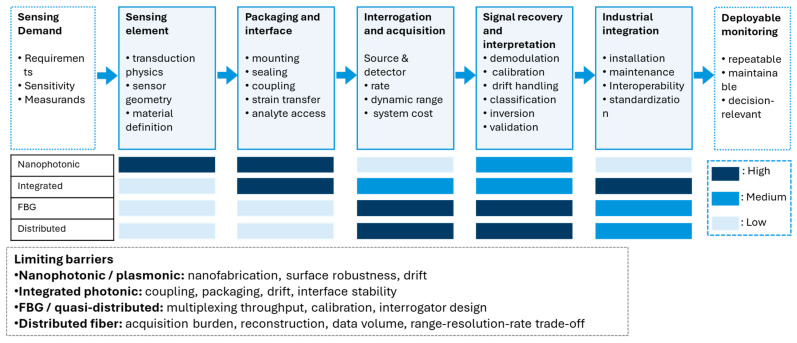
System-level deployment burden across optical sensing platforms. Burden levels are qualitative indicators derived from the reviewed implementations. High burden denotes an adoption-limiting constraint that must be addressed before reliable and cost-effective industrial deployment. Medium burden denotes a secondary engineering constraint affecting robustness, cost, or scalability. Low burden denotes a comparatively mature or manageable implementation step.

This system-level perspective is important because different optical platforms are not limited by the same bottleneck. Figure 12 qualitatively summarizes where deployment constraints accumulate across the optical sensing chain. A high burden denotes an adoption-limiting constraint that must be addressed before the platform can progress toward reliable and cost-effective industrial deployment. A medium burden denotes a secondary but still relevant engineering constraint that affects robustness, cost, or scalability, while a low burden indicates a comparatively mature or manageable implementation step. The assignments were derived from the reviewed implementations by considering sensing-interface stability, packaging and environmental protection, optical coupling interface, interrogation-hardware complexity, calibration drift, data processing load, installation constraints, and compatibility with current industrial maintenance processes. Within this framework, nanophotonic and plasmonic sensors are mainly limited by surface stability, fouling, and fabrication repeatability; PIC sensors by coupling, packaging, drift, and interface robustness; FBG and quasi-distributed systems by multiplexing, calibration, and interrogator throughput; and distributed fiber sensors by acquisition burden, reconstruction complexity, data volume, and the range-resolution-rate trade-off. A higher burden therefore indicates a larger unresolved system-level gap between sensor demonstration and stable industrial deployment. Industrial adoption is consequently governed by the weakest system link, not only by sensor sensitivity.

### 8.1. System-Level and Interrogation Challenges

A major barrier to the broader industrial deployment of optical sensing is the interrogation unit. In distributed fiber-optic architectures, systems based on OTDR, OFDR, Raman, and Brillouin scattering depend heavily on narrow-linewidth lasers, high-speed acquisition, coherent detection, and extensive signal processing [5]. Since the sensing fiber is inexpensive and often easy to install, the interrogator usually sets the system cost, footprint, and power budget, especially when long range, high spatial resolution, and fast update rate are required at the same time. Performance in these distributed architectures is governed by physical trade-offs among sensing reach, spatial resolution, update rate, and signal-to-noise ratio (SNR). Under standard performance benchmarks, such as sustaining over 30 km of range with 1 m spatial resolution, 1 K temperature uncertainty, and 1 to 60 s acquisition times, capabilities are ultimately constrained by system-level SNR [48]. Attempts to bypass these limits often shift the engineering burden rather than eliminate it. Optical amplification extends reach and improves detectability, but introduces dynamic-range penalties, additional noise, and architectural complexity [6]. Similarly, highly sensitive detection schemes reduce the need for amplification in low-signal regimes, but impose timing and integration constraints on the host electronics [193].

However, this interrogation burden is no longer permanently tied to benchtop instrumentation. Photonic integration successfully transfers readout complexity into compact hardware, as demonstrated by monolithically integrated SOI AWG-photodetector interrogators that achieve 6.79 pm accuracy and 1 pm wavelength resolution for continuous FBG readout [75]. From a deployment perspective, the critical architectural distinction is not between fiber-based and chip-based sensors, but between systems requiring complex coherent distributed readout and those based on the spectral tracking of localized optical features. For PIC and nanophotonic sensors based on resonance or spectral-shift readout, the interrogation burden can partly overlap with established FBG practice, because both require stable wavelength referencing, peak tracking, and compensation of drift across multiplexed sensing channels [194].

### 8.2. Integration and Packaging Challenges

An obstacle in multi-scale optical sensing is that system integration often becomes more difficult than demonstrating the sensing principle. Device-level performance must survive the cumulative stresses of coupling, packaging, mounting, encapsulation, and prolonged operation under industrial conditions. The viability of most sensing concepts is defined by the ability to maintain mechanical stability and optical efficiency within an environmentally robust assembly [21].

The critical failure points occur at platform interfaces, specifically in fiber-to-chip interconnection. Performance and long-term reliability are governed by alignment tolerances, coupling losses, mechanical stability, and thermal-expansion mismatches between different materials [195]. For integrated and nanophotonic sensors, packaging can be seen as an active functional component, affecting coupling efficiency, baseline drift, and calibration stability. Therefore, successful deployment requires treating photonic integrated circuit (PIC) packaging as a primary manufacturing requirement at the component, chip, and system levels simultaneously [196].

Industrial survival requires sensors to maintain accuracy under severe operating conditions, including extreme thermal cycling, vibration, and strain transfer through mounting structures. Furthermore, chemical exposure and the physical embedding processes often alter device responses in ways that are not captured during laboratory calibration. While fiber-optic architectures benefit from relatively mature deployment and protection strategies, integrated and nanoscale photonic sensors still lack the standardized methodologies required for robust encapsulation and reproducible field installation. Industrial viability depends on matching robustness, embeddability, interrogation burden, and power or connectivity requirements to the constraints of the specific machine, asset, or process [197].

Beyond the device itself, installation and maintenance workflows present a significant constraint. Optical sensing systems are frequently deployed within infrastructures designed for conventional electrical cabling. In these environments, system reliability is determined by fiber routing, bend-radius control, connector cleanliness, and mechanical strain relief [198]. As a result, the longevity of an industrial sensing network depends mainly on the robustness of these handling and packaging interfaces.

**Figure 13:**
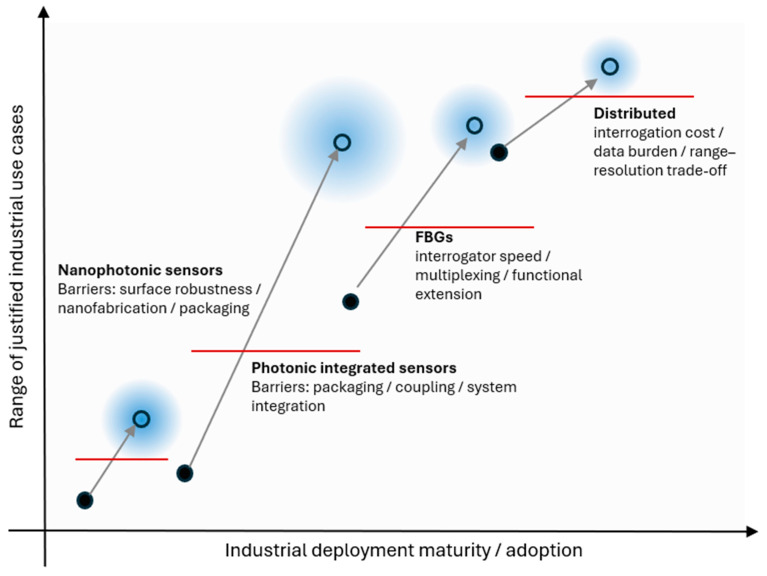
Qualitative deployment trajectories of major optical sensing platforms in industrial monitoring. Filled markers indicate current deployment maturity, open markers indicate projected positions, blue regions denote the plausible future deployment space, and arrows show expected expansion if the dominant platform-specific bottlenecks (red lines) are reduced.

Figure 13 summarizes the expected deployment evolution of the main optical sensing platforms using two qualitative axes: industrial deployment maturity/adoption and scope of industrial applicability. The horizontal axis reflects the present level of industrial adoption, including field validation, availability of interrogation hardware, packaging maturity, installation practice, and integration with industrial monitoring workflows. The vertical axis reflects the range of industrial sensing problems for which each platform has a technically justified role, rather than its nominal sensitivity or scientific novelty. The arrows indicate plausible deployment expansion if platform-specific barriers are reduced, not guaranteed adoption trends. Distributed fiber and FBG/quasi-distributed systems already occupy the more mature part of the industrial landscape because their installation routes, interrogation methods, and field use cases are comparatively established. Integrated photonic sensors are positioned at lower current maturity but may expand as coupling, packaging, and drift-control methods become more robust. Nanophotonic and plasmonic sensors remain the most application-specific, with future growth depending on surface stability, fouling control, and reproducible fabrication. The required transition is therefore larger for integrated and nanoscale photonic platforms than for fiber-based systems, which mainly require refinement of interrogation cost, multiplexing, field qualification, and integration with industrial data infrastructures rather than proof of deployability. Adoption should therefore be treated as a system-level problem, where future industrial use is governed by whether each platform can overcome its main implementation barriers.

### 8.3. Data Quality and Model Reliability Challenges

The industrial viability of machine-learning (ML) methods in optical sensing is determined by data quality and model architecture. Industrial environments generate optical signals characterized by drift, installation-induced variability, sensor aging, and process-dependent fluctuations. Because representative, labeled datasets for these non-ideal conditions are scarce and costly to acquire [199], data quality must be treated as a sensing constraint. Also, measured signals shift while changing boundary conditions, operational regime transitions, and environmental conditions. This operational variability is a recognized obstacle to reliable damage identification and model validation [200,201]. Consequently, models trained on controlled datasets frequently lose predictive reliability when transitioned into full-scale production.

Industrial adoption remains conservative because manufacturing processes cannot be adjusted on the basis of benchmark accuracy alone. ML-assisted optical sensing must be validated under the operating conditions in which production decisions are made, including installation-dependent coupling, thermal history, process transients, environmental drift, and changes in the monitored asset. A model that performs well on curated laboratory data is not deployment-ready unless its uncertainty, failure modes, and transferability have been tested within the relevant production envelope [2]. Although deep-learning methods are increasingly reported for SHM and fiber-optic sensing, the main unresolved issue is still deployment-grade validation [199,202].

The reliability problem also extends to the sensing layer itself. As FBG and quasi-distributed networks scale, anomalous wavelength shifts can originate from the monitored structure, but also from sensor debonding, connector degradation, channel crosstalk, or demodulation errors. WDM/SDM architectures require channel-level diagnostics, anomaly indices, and demodulation quality checks to separate asset changes from degradation of the sensing chain [203].

### 8.4. Industrial Deployment and Standardization

Scalability, standardization, and total cost define the upper-level limits of industrial adoption. Practical integration depends on whether the full sensing architecture, including interrogation, housing, installation, and data processing, can be manufactured, deployed, and maintained within existing industrial workflows at acceptable cost and complexity [1]. In fiber-optic architectures, long sensing range is only one part of scalability. Wider coverage, finer spatial resolution, and faster update rates place most of the burden on the interrogator, the acquisition hardware, and the processing of large data streams [5]. Moreover, while wafer-level fabrication and sub-millimeter footprints make integrated and nanophotonic sensors attractive, their industrial scale-up is related to reproducible packaging, stable long-term calibration, and standardized system-level interfacing [196].

Industrial cost should be evaluated at the system level, not only at the price of the sensing element. Packaging, installation, maintenance, and compatibility with existing monitoring equipment often determine whether a sensing system is adopted [194]. The current absence of standardized platforms, unified interfaces, and established qualification routes forces each sensing solution into a cycle of costly customization, which slows industrial uptake. Thus, wider deployment depends on the transition toward sensing architectures that are modular, interoperable, and maintainable under realistic operating conditions, and become scalable and economically feasible [1].

A partial route toward standardization already exists through optical-sensor-specific standards, although these standards do not yet remove the need for application-specific packaging and installation qualification. The IEC 61757 series provides the most relevant framework for fiber-optic sensors, defining generic specifications, terminology, and performance characterization methods for fiber-optic sensor systems and their subassemblies [204]. For distributed acoustic and vibration sensing, IEC 61757-3-2 specifies terminology, characteristic performance parameters, test and calculation methods, and test equipment for interrogation units based on Rayleigh backscatter and phase-sensitive coherent OTDR [205]. In parallel, IEEE Std 3101-2023 defines critical DAS-interrogator terminology and performance parameters so that DAS systems can be described and compared more consistently across suppliers [206]. These standards can improve comparability at the interrogator and sensor-system level, but they do not fully standardize bonding geometry, strain-transfer validation, encapsulation, field installation, or maintenance routines. Therefore, industrial standardization of optical sensing will likely require two coupled layers: sensor-performance standards such as IEC 61757 and IEEE Std 3101, followed by application-specific qualification procedures for the mechanical package and installation method.

### 8.5. Outlook and Strategic Directions

Future progress in optical manufacturing sensing will depend less on isolated gains in device sensitivity than on the successful integration of hardware, interrogation, packaging, and data interpretation into robust, field-ready industrial systems [2]. The central challenge is no longer only the development of new sensing elements, but the delivery of multi-scale optical measurements that remain reliable, maintainable, and decision-relevant under industrial conditions.

The strongest advances will come from system-level co-design, where the sensing mechanism, interrogator, packaging route, and digital infrastructure are developed together. Distributed fiber sensing, integrated photonics, and nanophotonic platforms should therefore be treated as complementary sensing layers rather than competing solutions. Their industrial role will depend on standardized interrogation, robust packaging, validated predictive models, and digital-twin interfaces that can operate within real manufacturing workflows [207]. At the end, the value of optical sensing in manufacturing will be measured by whether the complete measurement chain can support repeatable industrial measurements.

## 9. Conclusions

Optical sensing in manufacturing is viewed as a multi-scale measurement infrastructure. Distributed fiber sensing, FBG and quasi-distributed networks, integrated photonic sensors, and nanophotonic devices operate over different spatial supports, sensing volumes, measurands, and deployment constraints, each one targeted for a specific sensing use case. The central conclusion is therefore not that optical sensing should replace conventional industrial instrumentation, nor that one optical platform is generally superior, but that each optical architecture becomes valuable only when its sensing geometry, observable, interrogation burden, and deployment constraints match the industrial measurement problem.

Fiber-optic systems remain the most established route for spatially extended monitoring, particularly when strain, temperature, vibration, acoustic activity, or leakage must be tracked over large assets or embedded structures. Integrated photonic sensors target compact, multiplexable, component-level monitoring, but their industrial use still depends on coupling, packaging, drift control, and interface stability. Nanophotonic and plasmonic sensors provide highly localized and surface-sensitive transduction for chemical, interfacial, and nanoscale process signatures.

Across all platforms, the decisive constraint is the integrity of the full measurement chain. A sensing solution becomes industrially useful when transduction, packaging, interrogation, calibration, signal recovery, data interpretation, and model coupling remain stable under the relevant industrial conditions. Further progress will therefore come from system-level co-design that turns optical observables into maintainable and repeatable measurements. This shift from sensing demonstration to qualified monitoring defines the practical role of optical sensing in industry and smart manufacturing.

## Figures and Tables

**Table 1:** Deployment-level comparison between conventional industrial and optical sensing technologies.

Conventional Sensor Family	Typical Industrial Role	Main Deployment Limitation	Optical-Sensing Advantage
Thermocouples/platinum resistance thermometers/thermistors [23]	Contact temperature sensing for products, tooling, air temperature, and equipment monitoring	Point-based and contact-dependent, calibration, drift, electromagnetic fields, and sensor-placement uncertainty affect dense or embedded monitoring	Distributed or multiplexed temperature mapping, embedded tooling/product monitoring, passive remote thermal readout
Electrical strain gauges/conventional electrical strain sensors [22]	Local strain or vibration-related strain measurement on accessible structural surfaces and submerged components	Wiring, electrical noise, electromagnetic/radio-frequency susceptibility, bonding quality, and environmental exposure limit robustness	Passive strain sensing with lower electrical noise, remote interrogation, multiplexed strain monitoring in submerged, embedded, or high-EMI environments
Thermal imaging/infrared thermography [22,23]	Non-contact surface-temperature monitoring of panels, tools, machines, and thermally exposed components	Line-of-sight requirement, emissivity and surface-condition dependence, response-time limits, and surface-only measurement	Embedded, internal, or distributed thermal monitoring where surface imaging cannot access the relevant temperature field
Accelerometers/piezoelectric vibration sensors [24]	Vibration monitoring for rotating machinery, bearings, motors, gearboxes, shafts, and predictive maintenance	Sensor mounting, local cabling/data transmission, reliable acquisition, and complex signal processing strongly affect diagnostic quality	Dynamic strain or vibration sensing close to fault sources, interrogation in confined, rotating, or electromagnetically noisy machinery, multiplexed remote readout
Acoustic-emission sensors [25]	Passive detection of crack growth, delamination, fiber breakage, impact, and damage events in composites and structures	Source localization requires multiple sensors and is complicated by anisotropy, wave attenuation, coupling, noise, and sensor placement	Fiber-optic acoustic-emission or grating-based sensing with electromagnetic immunity, higher-temperature operation, embedment, and coupling with strain/temperature monitoring
Laser-induced breakdown spectroscopy/Raman process spectroscopy [26,27]	Rapid elemental or molecular analysis for steel, coal, recycling streams, slags, molten materials, and high-temperature process media	Optical access, calibration transfer, matrix or sample inhomogeneity, plasma/thermal background, window fouling, and probe survivability	Fiber-delivered Raman or protected optical probes for remote, in situ, high-temperature process chemistry
Resistive/electrochemical/catalytic gas sensors [28]	Local gas detection for safety, leak monitoring, environmental control, chemical processing, energy systems, mining, and oil-and-gas facilities	Harsh environments degrade sensing materials, electrodes, electrolytes, catalysts, wiring, electronics, and packaging, drift, poisoning, humidity, radiation, and corrosion reduce stability and selectivity	Remote or non-contact gas detection, isolation of sensitive electronics from the process zone, spectroscopic selectivity in high-temperature, corrosive, radiative, or inaccessible environments

**Table 2:** Comparative overview of major fiber-optic sensing architectures, which differ in optical observable, dominant measurand class, spatial regime, and interrogation burden.

Sensing Architecture	Optical Observable/Principle	Dominant Measurands	Spatial Regime	Typical Range/Resolution Character	Interrogation Burden	Representative Refs.
Fiber Bragg grating (FBG)/quasi-distributed	Bragg wavelength shift from periodic refractive-index modulation	Strain, temperature; secondary quantities via transduction or coatings	Point to quasi-distributed	Discrete sensing nodes; dense WDM multiplexing possible, but no continuous spatial profile	Low–medium	[22,29,30,31,32,33,38,47]
Rayleigh distributed sensing	Elastic backscatter/local optical-path perturbation; OTDR or OFDR interrogation	Strain, temperature, vibration/acoustic activity	Fully distributed	Highest spatial discrimination; generally shorter practical range than Raman/Brillouin in high-resolution implementations	High	[5,6,39,44,48]
Raman distributed sensing	Stokes/anti-Stokes backscatter ratio	Temperature	Fully distributed	Long-range thermal profiling; lower multifunctionality than Rayleigh/Brillouin architectures	Medium	[14,45,48]
Brillouin distributed sensing	Brillouin frequency shift from light–acoustic interaction	Strain and temperature	Fully distributed	Long-range thermomechanical monitoring; typically lower spatial resolution than high-resolution Rayleigh approaches	High	[15,46,48]

**Table 3:** Representative implementations of fiber-optic sensing in industrial monitoring.

Architecture	Deployment	Measured Quantity	Reported Performance	Ref.
FBG	Embedded lithium-ion battery monitoring	Internal temperature	Internal temperature differentials up to 8.6 °C under abnormal loads	[29]
FBG	70 MPa type IV hydrogen composite pressure vessel	Strain/structural state	In situ monitoring beyond 172.4 MPa burst pressure, strains up to 18,000 με, 100% sensor survival	[34]
FBG	Ship-bottom pressure-distribution monitoring	Pressure	Sensitivity of 58.94 pm/kPa and precision of 1.7 Pa	[35]
FBG	WO_3_–Pd-coated side-polished hydrogen sensor	Hydrogen concentration	Detection range of 0.5–12,000 ppm with 0.9 s response time at 4000 ppm	[36]
Rayleigh/distributed vibration sensing	Underground pipeline abnormal-event monitoring	Vibration/abnormal-event localization	Precise localization achieved through combined time- and frequency-domain feature extraction	[39]
Raman DTS	Field trial for oil-leakage detection in soil	Distributed temperature	Monitoring over 59 km with 2 m spatial resolution and 3.9 °C accuracy within 330 s	[45]
Brillouin/BOTDR	Oil-pipeline leakage monitoring via thermomechanical response	Leak detection	Leak detection at 1.1 m^3^/h, improved to 0.1 m^3^/h with plastic-film wrapping	[46]
FBG interrogation	Wavelength-to-time mapping with Gaussian filters	Wavelength demodulation	Interrogation rate of 264 MHz with wavelength error below 20 pm	[47]

**Table 4:** Summary of major integrated photonic platforms for sensing. The main PIC material platforms differ primarily in confinement, loss, active-functionality support, and mechanical compliance.

Platform	Main Strength	Best Fit in Sensing	Main Constraints	Ref.
Silicon photonics	Dense integration, CMOS compatibility, strong confinement	Compact resonant/interferometric sensing with high multiplexing density and electronic co-integration	Process sensitivity, higher propagation loss than SiN	[17,49,50]
Silicon nitride (SiN)	Low loss, broad transparency window, high-Q operation	Spectrally stable sensing, narrow-linewidth/low-noise readout, applications prioritizing fidelity over absolute miniaturization	Lower compactness than high-index silicon, not mature packaging	[17,52]
Indium phosphide (InP)	Active elements fabricable	Fully integrated systems requiring on-chip lasers, modulators, or detectors	More specialized fabrication ecosystem and higher integration complexity	[51]
Polymer photonics	Mechanical compliance, tunable thermo-mechanical response	Bounded surfaces, lightweight embedded layers, multi-axial, flexible sensing	Lower long-term thermal stability, lower confinement factor, large circuits	[53]

**Table 5:** Measurand-driven summary of integrated photonic sensing architectures.

Measurand	Main PIC Architecture	Indicative Result	Deployment Value	Key Limitation	Ref.
RI/absorption	Waveguides, rings, interferometers, functionalized cavities	5000 ppm CO_2_; on-chip H_2_/CO_2_ sensing	Compact gas/fluid/process-state nodes	Selectivity, fouling, drift	[54,55,56,57,58]
Temperature	Rings, gratings, thermo-optic waveguides	83.4 pm/°C	Localized thermometry, thermal referencing	Thermal drift remains intrinsic	[59,60,61,62]
Pressure/displacement	MZI, AWG, optomechanical/membrane-coupled devices	45 fm/Hz^1/2^ displacement imprecision, 40 MPa pressure capability	Local mechanically coupled sensing	Strong dependence on transducer and package design	[63,64,65]
Vibration/acceleration	Photonic MEMS, SOI MOEMS, inertial PICs	1274 Hz, up to 7 g; 21.80–32.47 ng/Hz^1/2^	Compact inertial/dynamic diagnostics	Packaging and interfacing burden	[66,67,68]

**Table 6:** Application-domain deployment logic of optical sensing platforms in smart manufacturing.

	Sensing Problem	Optical Architecture	Spatial Regime	Main Deployment Constraint
Infrastructure and energy systems	Monitoring of pipelines, power cables, overhead transmission corridors, utility-scale assets, and selected high-value nodes	Distributed optical fiber sensing: Raman, Brillouin, Rayleigh, FBGs at cable terminations, joints, valve stations, and conductor hotspots	Continuous spatial coverage over long linear assets, localized point sensing at selected hotspots	Placement, endpoint access, optical power budget, environmental exposure, field robustness
Structural health and component-level diagnostics	Monitoring of composite laminates, bonded joints, shell-like structures, pressure vessels, hydrogen storage tanks	Embedded or surface-mounted FBG arrays, distributed fiber sensing for strain mapping; integrated photonics only as localized add-ons	Quasi-distributed or distributed strain/pressure mapping across bounded structures, interfaces, and transition zones	Sensor survivability, strain-transfer fidelity, temperature-strain decoupling, embedment, packaging of localized photonic sensors
Manufacturing systems and machinery monitoring	Diagnostics of bearings, rotating shafts, gearboxes, electric-machine coils, robotic manipulators, heavy machine-tool bases	FBG arrays as the mature route, optical MEMS and integrated photonic inertial sensors as localized complements	Local sensing close to fault sources, rotating components, internal thermal regions, robotic joints, tactile surfaces	High-speed interrogation, fiber routing in moving assemblies, bonded-fiber fatigue, in situ calibration, and robust packaging/interfacing
Advanced manufacturing processes	Harsh environment monitoring, in situ spectroscopic monitoring, in-process monitoring of additive manufacturing, composite curing, resin flow, residual stress, tool-part interaction, process-state evolution	Embedded FBGs, long-gauge FBG/OFDR, distributed optical backscatter reflectometry, tool integrated PIC sensors	Process-zone, embedded material-state monitoring, tool-level sensing	Optical access, emissivity/calibration, embedded-sensor survivability, coupled temperature/pressure/refractive-index response, PIC packaging
Nanoscale and chemical sensing applications	Detection of local gas composition, hazardous species, isotope-specific gases, interfacial chemistry, ultrathin films, catalyst surfaces, adsorption layers, and contamination films	PIC absorption sensors, plasmonic sensors, catalytic-plasmonic sensors, suspended nanophotonic waveguides, dielectric metasurfaces, and metasurface microspectrometers	Highly localized chemical, interfacial, and surface-confined sensing at compact process nodes or critical interfaces	Chemical selectivity, humidity/temperature compensation, receptor aging, surface contamination, reproducible nanofabrication, analyte-access packaging

**Table 7:** Data interpretation in optical monitoring by linking optical inputs to processing tasks, derived outputs, and industrial constraints.

Computational Layer	Optical Input	Interpretation Task	Output	Industrial Constraint
DSP	spectra, phase, intensity, backscatter traces	demodulation, denoising, feature tracking	stable optical observables	latency, hardware burden
ML	multivariate/coupled responses	inverse mapping, classification, compensation	estimated measurand or process state	training data, drift, transferability
Sensor fusion	distributed + point + localized channels	reconcile non-equivalent measurements	fused diagnostic indicator	synchronization, uncertainty
Digital twin coupling	sensor-derived state variables	model-constrained updating	actionable state estimate	validated model, update rate

## Data Availability

No new experimental data were generated in this review. The PRISMA-style flow diagram and completed PRISMA 2020 checklist are provided as Supplementary Materials.

## Supplementary Materials

The following supporting information can be downloaded at: https://www.mdpi.com/article/10.3390/s26113581/s1, Figure S1: PRISMA-style literature-selection flow diagram describing the identification, screening, eligibility assessment, and inclusion process used for this deployment-oriented narrative review. The final set of studies was retained as representative technical evidence. Table S1: PRISMA 2020 checklist [208].

## Abbreviations

The following abbreviations are used in this manuscript:

AWG	Arrayed Waveguide Grating
BOTDR	Brillouin Optical Time-Domain Reflectometry
CMOS	Complementary Metal–Oxide–Semiconductor
COPV	Composite Overwrapped Pressure Vessel
CT	Computed Tomography
DAS	Distributed Acoustic Sensing
DFOS	Distributed Fiber-Optic Sensing
DSP	Digital Signal Processing
DTS	Distributed Temperature Sensing
FBG	Fiber Bragg Grating
FEM	Finite-Element Method
FPGA	Field-Programmable Gate Array
InP	Indium Phosphide
LENS	Laser-Engineered Net Shaping
LNG	Liquefied Natural Gas
LOD	Limit of Detection
LPG	Liquefied Petroleum Gas
LPBF	Laser Powder Bed Fusion
LSPR	Localized Surface Plasmon Resonance
MEMS	Micro-Electro-Mechanical Systems
MOEMS	Micro-Opto-Electro-Mechanical Systems
MOF	Metal–Organic Framework
MPW	Multi-Project Wafer
MZI	Mach–Zehnder Interferometer
OFDR	Optical Frequency-Domain Reflectometry
OTDR	Optical Time-Domain Reflectometry
PDMS	Polydimethylsiloxane
PIC	Photonic Integrated Circuit
PTAT	Proportional to Absolute Temperature
RI	Refractive Index
RMSE	Root Mean Square Error
SCADA	Supervisory Control and Data Acquisition
SHM	Structural Health Monitoring
SiN	Silicon Nitride
SNR	Signal-to-Noise Ratio
SOI	Silicon-on-Insulator
SPR	Surface Plasmon Resonance
TDM	Time-Division Multiplexing
VaRTM	Vacuum-Assisted Resin Transfer Molding
VOC	Volatile Organic Compound
WDM	Wavelength-Division Multiplexing
XLPE	Cross-Linked Polyethylene
