# Supplementary figures and images for: Review of Optical Fiber and Integrated Photonic Sensors for Industry and Smart Manufacturing: Technologies, Applications, Structural Health Monitoring and AI-Enabled Sensing
Source: Sensors (Basel). 2026 Jun 4;26(11):3581. doi: 10.3390/s26113581 (PMC13259292; doi:10.3390/s26113581)

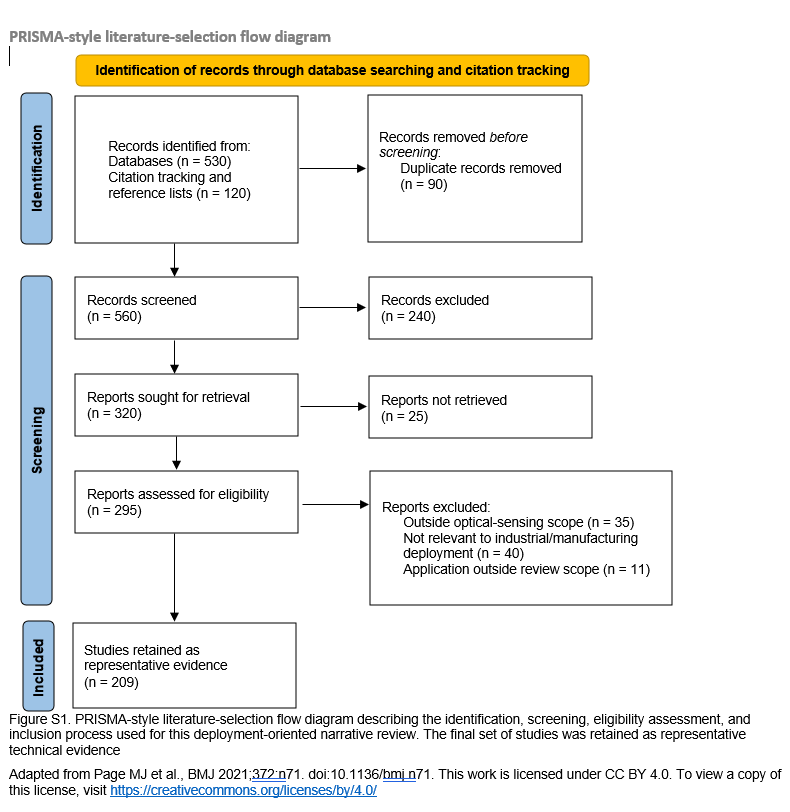

Supplement: Supplementary file 1 [file sensors-26-03581-s001.zip › Figure S1.png]
